# Electrochemically synthesized polymers in molecular imprinting for chemical sensing

**DOI:** 10.1007/s00216-011-5696-6

**Published:** 2012-02-03

**Authors:** Piyush S. Sharma, Agnieszka Pietrzyk-Le, Francis D’Souza, Wlodzimierz Kutner

**Affiliations:** 1 Department of Physical Chemistry of Supramolecular Complexes, Institute of Physical Chemistry, Polish Academy of Sciences, Kasprzaka 44/52, 01-224 Warsaw, Poland; 2 Faculty of Mathematics and Natural Sciences, School of Science, Cardinal Stefan Wyszynski University in Warsaw, Wóycickiego 1/3, 01-815 Warsaw, Poland; 3 Department of Chemistry, University of North Texas, 1155 Union Circle, # 305070, Denton, TX 76203-5017 USA

**Keywords:** Electrochemically synthesized polymer, Electronically conducting polymer, Molecular imprinting, Chemical sensor, Molecular recognition

## Abstract

This critical review describes a class of polymers prepared by electrochemical polymerization that employs the concept of molecular imprinting for chemical sensing. The principal focus is on both conducting and nonconducting polymers prepared by electropolymerization of electroactive functional monomers, such as pristine and derivatized pyrrole, aminophenylboronic acid, thiophene, porphyrin, aniline, phenylenediamine, phenol, and thiophenol. A critical evaluation of the literature on electrosynthesized molecularly imprinted polymers (MIPs) applied as recognition elements of chemical sensors is presented. The aim of this review is to highlight recent achievements in analytical applications of these MIPs, including present strategies of determination of different analytes as well as identification and solutions for problems encountered.

## Introduction

**Fig. 1:**
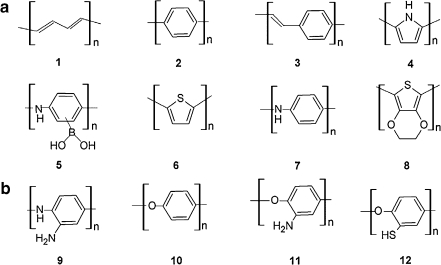
Structural formulas of the most common polymers prepared by electropolymerization. **a** Electronically conducting polymers: polyacetylene **1**, polyphenylene **2**, polyphenylenevinylene **3**, polypyrrole **4**, poly(aminophenylboronic acid) **5**, polythiophene **6**, polyaniline **7**, and polyethylenedioxythiophene **8**. **b** Electronically nonconducting polymers: polyphenylenediamine **9**, polyphenol **10**, polyaminophenol **11**, and polythiophenol **12**

Electroactive monomers can polymerize to form either conducting (Fig. 1a) or nonconducting (Fig. 1b) polymers. Among conducting polymers, the major group consists of electronically conducting polymers (ECPs).

Since the first synthesis of ECPs [1, 2], these polymers have been extensively studied for various purposes. ECPs are polymers with electronic properties (magnetic, conducting, and optical) similar to those of metals; therefore, they are also referred to as “synthetic metals.” However, they retain properties of conventional organic polymers. Moreover, they are all fabricated in a similar way.

Several stable ECPs have been synthesized, including polyacetylene **1**, polyphenylene **2**, polyphenylenevinylene **3**, polypyrrole (PPy) **4**, poly(aminophenylboronic acid) (PAPBA) **5**, polythiophene (PTh) **6**, polyaniline (PANI) **7**, and polyethylenedioxythiophene (PEDOT) **8** (Fig. 1a) [3–9]. The starting materials for synthesis of these ECPs include not only derivatives of hydrocarbons and heterocycles but also relatively novel monomers, such as 3,4-ethylenedioxythiophene (EDOT) [10, 11], whose electropolymerization results in ECPs of largely improved properties.

Because of their unique electrochemical properties, ECPs have attracted considerable interest in the development of chemical sensors and biosensors. According to the IUPAC recommended definitions, chemosensors [12] and biosensors [13] are integrated receptor–transducer devices in which a chemical or biological recognition unit, respectively, provides selective quantitative or semiquantitative analytical information. In other applications, for instance, in the construction of the polymer light-emitting devices [14–17], ECPs have already approached the stage of commercial display modules and organic light-emitting diodes. Moreover, electrophosphorescent light-emitting devices have been fabricated [18]. Other application examples include the design of polymer oxide batteries [19], redox supercapacitors [20, 21], and superconductors [22]. Furthermore, ECPs were used as ion-gate membranes for controlled release of anionic drugs [23], and as electrochemically switchable ion exchangers for water purification [24].

ECPs can be synthesized either chemically (by treatment with an external reactant) or electrochemically (by electropolymerization). Chemical synthesis of ECPs mostly involves monomers, which polymerize after oxidation. Therefore, this synthesis is performed by applying an oxidant, e.g., (NH_4_)_2_S_2_O_8_ or FeCl_3_. Chemical synthesis is commonly used for preparation of ECPs in solutions or in bulk solids.

In contrast, electropolymerization is mainly used for deposition of ECP films on conducting substrates. Advantages of this procedure include the possibility to control (1) the rate of polymer nucleation and growth by proper selection of the electropolymerization parameters, (2) the film thickness by the amount of charge passed during film deposition, and (3) the film morphology by suitable selection of an appropriate solvent and supporting electrolyte. Electropolymerization is performed under galvanostatic, potentiostatic, or most commonly potentiodynamic conditions. The latter may result in the ECP matrix forming disordered spatial chains. Apart from that, the resulting polymer film may be in its charged or neutral state at the end of this polymerization because solvated counter ions efficiently enter and leave the film during its growth upon film charging and discharging, respectively. This counter ion ingress to and release from the film in the oxidation–reduction cycles causes periodic expansion and compression, respectively, of the film volume. This effect, initially generating film defects, is due to polymer swelling and shrinking, respectively, because of the solvent exchange. In contrast, ECPs prepared by potentiostatic or galvanostatic polymerization are well ordered, charged, and doped with counter ions for neutralization of this charge.

ECPs are well-established matrices used for fabrication of chemosensors [25, 26]. Several reviews have discussed in detail their role in chemical sensing [27–33]. Their use as molecular recognition units in chemosensors is a recently emerging and rapidly growing research area [34–44], particularly if ECPs are combined with molecular imprinting.

Molecularly imprinted polymers (MIPs) are synthetic materials that are prepared by copolymerization, in a porogenic solvent, of functional and cross-linking monomers in the presence of a template. This template can be similar to the target substance [45] or can be the target substance itself—the analyte—that the polymer should subsequently selectively recognize. During MIP synthesis, first, the monomers bearing suitable functionalities and template molecules are preorganized in solution, i.e., self-assembled, to form a complex by virtue of either covalent or noncovalent interactions between their complementarily binding functionalities. These complexes are preserved during formation of a cross-linked polymer network. After subsequent removal of the template, molecular cavities complementary in size, shape, and orientation of their binding sites to those of the template are left in the network. These cavities are then capable of selective binding of analyte molecules even in the presence of similarly structured interfering analogues [46]. This universal supramolecular chemistry concept of generating synthetic receptors has gained rapid and wide attention manifested in numerous applications [47].

The analogy between MIPs and biologically originatingreceptors as affinity reagents is straightforward. MIPs imitate the receptor–ligand, antibody–antigen, or enzyme–substrate biorecognition [48]; therefore, MIPs can be used in applications relying on selective molecular binding [46, 49]. Mechanical and chemical stability, ease of preparation, and the relatively low cost of MIP materials make them attractive for analytical applications involving column packings in chromatographic separations [50], sorbents for solid-phase microextraction [51, 52], and recognition units in chemical sensors [53–57].

Initially, MIPs were mostly prepared by free-radical polymerization, resulting in nonconducting polymers. This polymerization requires the presence of a polymerization initiator and light or heat to induce it. The resulting MIPs were tested for applications as recognition units of chemosensors utilizing different transduction schemes [58]. Most of the procedures for chemical synthesis of MIPs involved noncovalent preassembly in solution of the template and a vinylic or acrylic functional monomer followed by bulk polymerization [59]. MIPs prepared that way suffer from numerous deficiencies, as outlined below.

Several procedures are used to prepare MIP films directly on transducer surfaces, including (1) electropolymerization of an electroactive functional monomer, (2) drop-coating of a solution of a pre-prepared polymer, (3) preparation of composite membranes containing a conducting material (e.g., carbon nanotubes, graphite, or carbon black), MIP particles, and a binder (e.g., PVC), and (4) in situ chemical polymerization of a complex of a functional monomer and a template in solution which is then deposited by spin coating to form a thin film. In comparison with other procedures of MIP film preparation, such as drop-coating, composite-making, and spin-coating, the MIP films prepared by electropolymerization have superior properties with respect to adherence to the transducer surface as well as simplicity and speed of preparation. Moreover, the electropolymerization enables easy control of the film thickness and morphology as well as high reproducibility and the possibility of polymer preparation and operation in aqueous solutions [60].

Application of insulating acrylic or vinylic MIP films as recognition units of electrochemical sensors appeared difficult because of the lack of a direct path for electron conduction from the recognition sites to the electrode transducer; therefore, most of the chemosensors based on these films use either optical or gravimetric transduction. Hybrid materials combining these insulating MIPs with ECPs feature networks of molecular wires connecting recognition sites to the electrode surface [61]. MIP films prepared by free-radical polymerization with acrylic or vinylic monomers sometimes suffer from incomplete template removal during MIP preparation. A procedure of molecular imprinting using ECPs avoids this undesired effect. That is, the template can readily be removed from an MIP by film overoxidation [35]. Chemical-recognizing sites coming from moieties of the polymerized electroactive functional monomers are spatially positioned around the molecular cavity, formed to function as a permanent memory element for the imprinted template. Moreover, generally poor solubility and structural rigidity of ECPs contribute to their suitability as imprinted matrices as these features help to preserve the integrity of the imprinted sites after template removal.

**Fig. 2:**
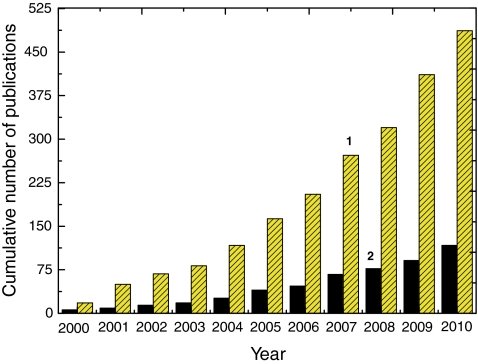
Cumulative number of articles published during the last decade in the field of chemical sensors using the concept of molecular imprinting: *1* all publications on molecularly imprinted polymer based chemical sensors and *2* publications on chemical sensors using molecularly imprinted polymers prepared by electropolymerization. (Data taken from SciFinder)

The number of publications on MIP based chemical sensors has grown exponentially over the last decade (histogram 1 in Fig. 2), and electrosynthesized polymers are still being more and more widely exploited for use in chemical sensors (histogram 2 in Fig. 2). PPy, PAPBA, PTh, PEDOT, PANI, poly(1,2-phenylenediamine), polyphenol, and polythiophenol are electroactive functional monomers which have been used extensively to devise chemosensors based on the concept of molecular imprinting. The first report on molecularly imprinted sensing was published in 1993 [58]. Since then, several MIP recognition and different transduction units have been explored for fabrication of chemical sensors. Herein, we highlight recent achievements in research on electrosynthesized MIPs for chemosensor applications, strategies of analyte determination developed, and identification and solution of problems encountered.

## Mechanism of electropolymerization

The most common electrochemical method for preparation of ECPs is anodic oxidation of suitable electroactive functional monomers; cathodic reduction is used much less frequently. In the former, formation of a polymer film and doping of counter ions as a result of oxidation occur simultaneously. Most often, the potential of monomer oxidation leading to polymerization is higher than that of charging of oligomeric intermediates or the resulting polymer. The mechanism of electropolymerization leading to the ECP is still incompletely understood and is discussed in different ways [62–67].

**Fig. 3:**
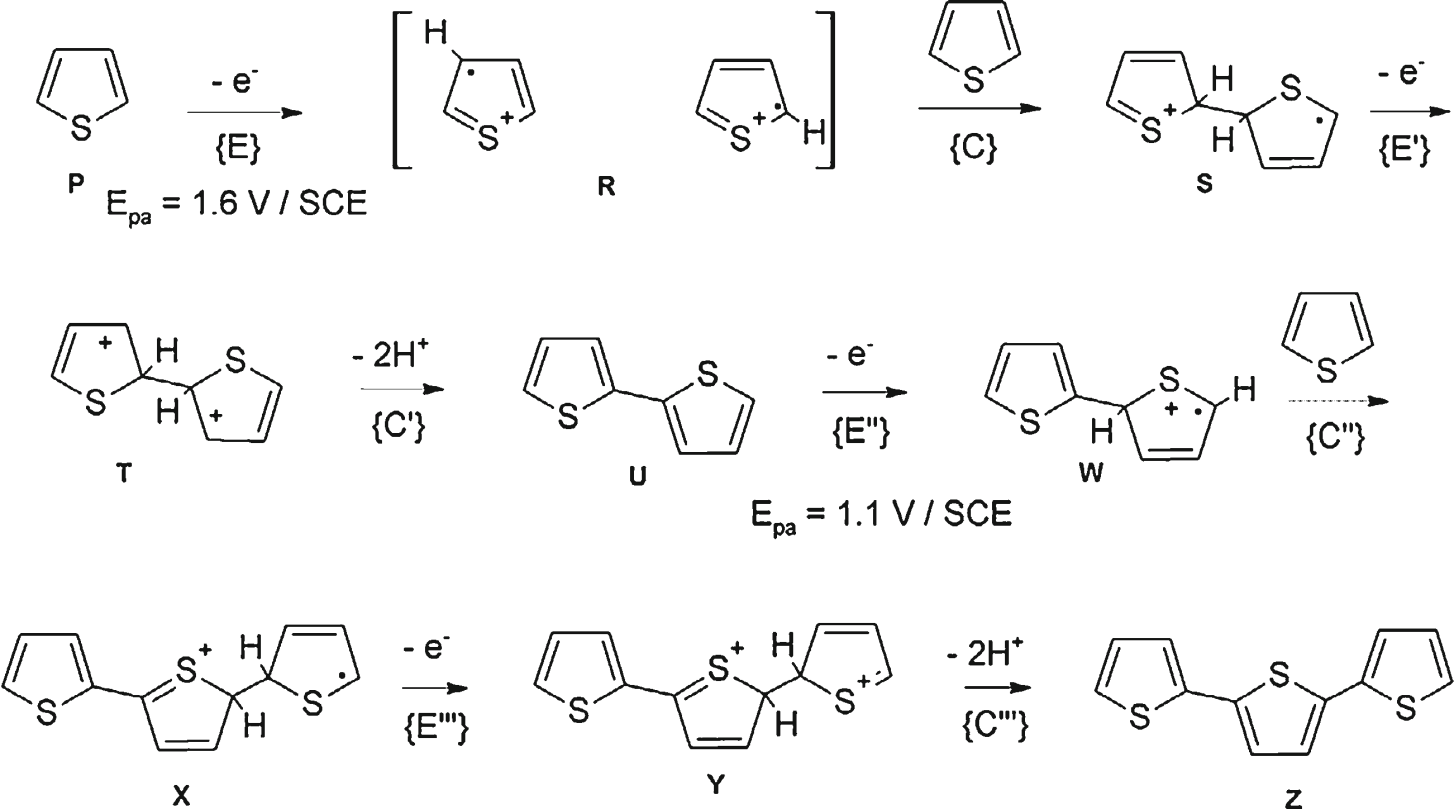
Basic mechanism of thiophene electropolymerization. SCE saturated calomel electrode. (Adapted from [68])

A simplified mechanism of electropolymerization of an electroactive monomer, such as pyrrole (Py) or thiophene (Th) involves alternate chemical {C} and electrode {E} reaction steps. For instance, in potentiodynamic electropolymerization of Th (Fig. 3) [68], a radical cation R is most likely formed in the first, {E} step of thiophene electrooxidation, manifested by an anodic peak of high positive potential (*E*_p,a_ = 1.6 V vs. the saturated calomel electrode, SCE). At the second, {C} step, R reacts with the monomer P and the protonated dimer of a radical cation S is formed. Next, S is electrooxidized to dication T (so-called doubly charged *σ*-dimer) at the {E’} step. A neutral dimer U is the product of elimination of two protons {C’}. Then, this dimer is oxidized in the second {E”} step, forming a radical cation W. In turn, W reacts with P in the {C”} step. These steps follow one another in accord with the ECE’C’… mechanism to form a trimer, Z. Advantageously, the dimer R is electrooxidized at a potential (*E*_p,a_ = 1.1 V vs. SCE) lower than that of electrooxidation of the monomer P.

## Origin of electrical conductivity of conducting polymers

In conventional nonconducting organic polymers such as polyethylenes, the valence electrons are bound in the *sp* ^3^-hybridized atomic orbitals engaged in covalent bonds. The mobility of such “*σ*-bonding electrons” is low and, therefore, these electrons do not contribute to the electrical conductivity of the polymer. The situation is completely different in ECPs. Contiguous *sp* ^2^-hybridized carbon atom centers form backbones of ECPs. One valence electron on each center resides in a *p*_*z*_ atomic orbital, which is orthogonal to three *σ* bonds formed by other *p* orbitals. These *p*_*z*_ electrons of adjacent centers interact to form conjugated *π* molecular orbitals. The electrons in these orbitals are delocalized. Moreover, they are highly mobile if the polymer is “doped” with counter ions by oxidation or reduction. This oxidation or reduction charges the polymer positively or negatively, respectively, by removing or injecting some of the delocalized electrons. Thus, the conjugated *π* orbitals form a one-dimensional electronic band and the electrons within this band become mobile when it is partially emptied [69–72].

## Electrosynthesized polymers in molecular imprinting

**Fig. 4:**
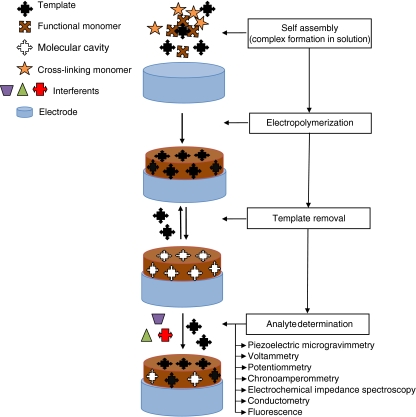
General procedure for molecular imprinting with an electroactive functional monomer and typical signal transduction methods employed in detection

For preparation of conducting or nonconducting molecularly imprinted thin polymer films directly on the transducer surface, electrochemical polymerization is a more and more frequently selected procedure [61, 73]. In this procedure, neither a polymerization initiator nor UV light or heat is needed. Moreover, an MIP film is deposited from a functional monomer solution of a porogenic solvent, sometimes with the use of a cross-linking monomer [40], directly onto an electrode surface in the presence of a template (Fig. 4). This film adheres well to a (roughened) electrode surface. The film thickness is governed by the amount of charge transferred during electropolymerization. Surface morphology is controlled by the selection of a suitable solvent and supporting electrolyte. Solvent swelling and inclusion of ions of a supporting electrolyte tunes the rigidity and porosity of this film. For electropolymerization, electroactive functional monomers containing electropolymerizing moieties, such as Py, 3-aminophenylboronic acid (APBA), Th, EDOT, metalloporphyrin, aniline (ANI), 1,2-phenylenediamine (PD), phenol (Ph), and 2-aminothiophenol (2-AThPh) (Fig. 1), are most often used.

### Electrosynthesized conducting polymers in molecular imprinting

Notably, the Py, APBA, PTh, and ANI functional monomers produce molecularly imprinted ECPs (MIECPs) after polymerization in the presence of a template. Template imprinting in these MIECPs can be facilitated by formation of hydrogen bonds between atoms of binding sites of the template and atoms of recognition sites of the MIECP. Additionally, *π*–*π* interactions between the MIECP and the template make the cavity more selective. That is, the presence of *π* electrons in the MIECP enhances stabilization of dispersive interactions between the aromatic ring of the functional monomer and the template. This stabilization is enhanced if a *π*-delocalized bond is involved, for example, in the *π*-conducting polymer chain. In this section, we discuss the use of different ECPs for preparation of MIPs and their application as recognition units in chemosensors.

#### Pyrrole-based functional monomers

Among different ECPs, PPy has been widely used for preparation of MIP systems because of its excellent biocompatibility and ease of immobilization of different biologically active compounds (Table 1). PPy is overoxidized at positive potentials, often regarded as undesired. That was because this overoxidation resulted in polymer degradation leading to the loss of conductivity and dedoping [74–82]. Despite these disadvantages, overoxidized PPy (OPPy) has been used in several electroanalytical applications.

**Table 1:** Analytical parameters of molecularly imprinted polymer (*MIP*) chemosensors using pyrrole as the functional monomer

Template/analyte	Transduction/electrode	Electropolymerization conditions	**S**olution for MIP preparation	Solution for analyte determination	Linear dynamic concentration range	LOD	Reference
Adenosine/inosine/ATP	Voltammetry/GCE	Potentiostatic +0.9 V vs. Ag/AgCl	0.1 M (TBA)ClO_4_/ACN	0.5 M PBS (pH 7.0)	-	-	[34]
			0.1 M (TBA)ClO_4_/methanol (80%)				
DS	PM/Pt	Galvanostatic at current density 1 mA cm^-2^	0.1 M Tris buffer (pH 9.0)	0.1 M Tris buffer (pH 9.0)	10 nM to 100 μM	-	[90]
Caffeine	PM/Au	Galvanostatic at current density 4 mA cm^-2^	0.1 M PBS (pH 7.0)	0.1 M PBS (pH 7.0)	0.1–10 mg mL^-1^	4.7 μg mL^-1^	[92]
Caffeine	Chronoamperometry/Pt	Potential pulses between 0.95 V (1 s) and 0.35 V (10 s) vs. Ag/AgCl	0.1 M phosphate buffer (pH 7.0)	0.1 M phosphate buffer (pH 7.0)	Up to 90 mM	-	[93]
Caffeine	Voltammetry/Au	Potential pulses between 0.3 V (1 s) and 0.7 V (10 s) vs. SCE	0.05 M KCl	0.1 M PBS (pH 7.0)	10–40 μM	-	[94]
Paracetamol	Voltammetry/PGE	Potentiodynamic −0.6 to +0.8 V vs. Ag/AgCl	0.1 M LiClO_4_	0.1 M KCl, PBS (pH 7.0)	5 μM to 0.5 mM	0.79 μM	[95]
					1.25–4.5 mM		
Sulfamethoxazole	Voltammetry/PGE	Potentiodynamic −0.6 to +0.1.4 V vs. Ag/AgCl	0.1 M (TBA)ClO_4_/ACN	BR buffer (pH 2.5)/50% ACN	0.025–0.75 mM	0.36 μM	[96]
					0.75–2.0 mM		
Ascorbic acid	Voltammetry/PGE	Potentiodynamic −0.6 to +0.8 V vs. Ag/AgCl	0.1 M LiClO_4_	0.1 M KCl, 0.05 M PBS (pH 8.5)	0.25–7.0 mM	74 μM	[97]
l-Asp	PM/Au	Galvanostatic at current density 10 μA cm^-2^	Aqueous (pH = 6.0)	0.05 KCl/HCl (pH–1.6)	-	-	[98]
			1 M NaOH (pH 11)				
			0.01 M NaPSS (pH = 6.0)				
Phenylalanine	Impedimetry, CD/Pt	Galvanostatic at current density 0.3 mA cm^-2^	0.04 M CSA/water	-	5–200 mg L^-1^	-	[99]
DCPA	Voltammetry/GCE	Potentiodynamic −1.3 to +1.0 V vs. Ag/AgCl	0.05 M PBS (pH 6.86),	0.1 M KCl	1–10 μM	0.83 μM	[102]
			0.1 M KCl				
Tryptophan	Voltammetry/Pt	Potentiostatic at +0.8 V vs. SCE	0.1 M NaCl (pH 6.5)	0.1 M NaCl	-	-	[103]
Zearalenone	SPR/Au	Potentiostatic at +0.9 V vs. SCE	0.2 M (TBA)BF_4_	0.1% ethanol	0.3–3,000 ng mL^-1^	0.3 ng g^-1^	[104]
TCAA	PM, CV/Au	Galvanostatic at current density 0.1 mA cm^-2^	0.25 M KCl	0.2 mM phosphate buffer (pH 7.0)	0.1–100 mg L^-1^	-	[105]
					0.1–1,000 mg L^-1^	-	
OTA	SPR/Au	Potentiostatic at +0.85 V vs. SCE	Ethanol/water (1:9, v:v)	Ethanol/water (1:9, v:v)	0.05–0.5 mg L^-1^	0.01 mg L^-1^	[107]
gp51	Chronoamperometry/Pt	Potential pulses between 0.95 V (1 s) and 0.35 V (10 s) vs. SCE	100 mM KCl	0.5 M KCl, 0.1 M phosphate buffer (pH = 7.2)	Up to 15 mg mL^-1^	-	[109]

*LOD* limit of detection, *DS* dodecyl sulfate, *l* *-Asp*, l-aspartic acid, *DCPA* 2,4-dichlorophenoxyacetic acid, *TCAA* trichloroacetic acid, *OTA* ochratoxin, *GCE* glassy carbon electrode, *PM* piezoelectric microgravimetry, *PGE* pencil-graphite electrode, *CD* circular dichroism, *SPR* surface plasmon resonance, *SCE* saturated calomel electrode, *TBA* tetrabutylammonium, *ACN* acetonitrile, *Tris* tris(hydroxymethyl)aminomethane, *PBS* phosphate-buffered saline, *NaPSS* sodium poly(styrene sulfonate), *CSA* camphorsulfonic acid, *BR* Britton–Robinson

In that direction [34, 83], molecularly imprinted OPPy (MIOPPy) ultrathin films were grown on glassy carbon electrode (GCE) surfaces for determination of adenosine, inosine, and adenosine 5′-triphosphate (ATP). Apparently, templating with these purines and the nucleotide affected the selectivity and sensitivity of the MIOPPy films. Moreover, templating with ATP via its electrostatic interactions with the positively charged PPy most significantly influenced the response of the MIOPPy films. The films were deliberately made thin to ensure high permeability. That way, problems with detectability, which are common for thicker films, were eliminated and the chemosensor response was controlled by the analyte–film interactions rather than by the rate of analyte transport through the film.

Moreover, OPPy has been used for imprinting of amino acids [35, 84–88] and bile acids [89]. For instance, a molecularly imprinted PPy (MIPPy) film templated with the L-glutamate neurotransmitter was deposited by galvanostatic electropolymerization and this was followed by overoxidation to dedope the L-glutamate template [35, 86]. This MIOPPy film was capable of enantioselective recognition of the cationic L-glutamic acid (L-GA), as examined by cyclic voltammetry (CV), piezoelectric microgravimetry (PM) using an electrochemical quartz crystal microbalance, and fluorimetry with an uptake ratio of the L-to-D enantiomer exceeding 10. The MIOPPy film doped with D-glutamate was selective for D-glutamate [86]. Then, as further extension of this work, an L-alanine template was enantioselectively captured with other MIPPy forms, such as colloidal MIPPy particles [85]. Apparently, OPPy is promising as a versatile, highly selective molecular recognition matrix advantageously requiring just a straightforward synthesis. For imprinting of target amino acids, L-lactate was used as a replacement template because of difficulties in preparing the PPy film doped with the target amino acid as the template. In fact, out of 21 protein amino acids, only GA and aspartic acid (Asp) can be used as templates. This is because other amino acids are not dissociated and, therefore, do not carry the negative charge in neutral and acidic solutions in which Py is electropolymerized. However, the rate of formation of MIOPPy for determination of glutamate or aspartate was low in this case, indicating that neither of these amino acids was in fact suitable as a template. To overcome this problem, a lactate structural analogue was used as the template instead for preparation of an MIP for determination of alanine [84, 87, 88]. In other work using an MIOPPy colloid templated with L-lactate, affinity for L-alanine was higher than that for D-alanine and the L-to-D enantiomer uptake ratio was as high as 11. Furthermore, a 1-naphthalenesulfonate (1-NS)-templated MIOPPy colloid was prepared for determination of 1-NS. As expected, this colloid absorbed 1-NS better than 2-naphthalenesulfonate. Evidently, the shape-complementary cavity formed on the colloid surface precisely recognized the difference in the spatial configuration of alanine enantiomers and naphthalenesulfonate structural isomers [88].

Further, dodecyl sulfate (DS) was templated in a galvanostatically synthesized MIPPy film to prepare a recognition unit of the PM chemosensor [90]. The DS template was then removed with distilled water. The detectability was better and the linear concentration range was wider for this PM chemosensor than for the potentiometric chemosensor for DS (Table 1) [91].

A PM caffeine chemosensor was prepared by direct galvanostatic deposition of an MIPPy film onto the Au electrode of a quartz resonator [92]. After electropolymerization, the caffeine template was extracted with water. The linearity and detectability of the chemosensor fabricated at low (4 mA cm^-2^) current density were favorable; however, at high (6 mA cm^-2^) current density those parameters were very limited owing to the formation of a thicker sensing film with less accessible imprinted cavities. Unfortunately, the chemosensor response was linear in a very narrow concentration range (Table 1). Moreover, this response was affected by the presence of interferents with molecular structure similar to that of caffeine, particularly theophylline and xanthine.

Furthermore, Py has been used for preparation of a chronoamperometric chemosensor for caffeine [93]. The binding affinity of the recognition sites of the imprinted cavities was sufficient to detect caffeine. Elution and binding of caffeine to this MIPPy film was detected by pulse chronoamperometry (Table 1).

**Fig. 5:**
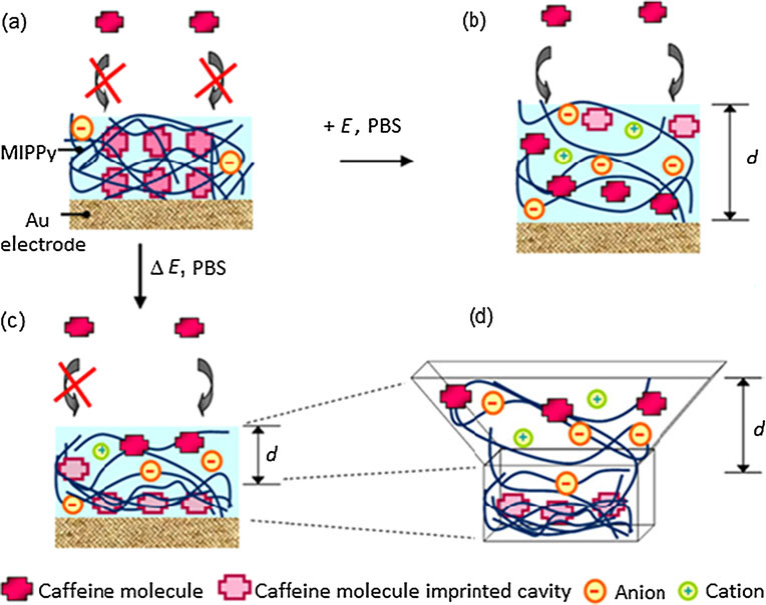
Illustration of the mechanism of alteration of the effective thickness, *d*, of a molecularly imprinted polypyrrole (*MIPPy*) polymer film with electrical potential stimuli. *a* at equilibrium, the film is not swollen and ingress of caffeine molecules is precluded. *b* upon application of a positive potential, the polymer swells and opens up all the imprinted cavities for caffeine binding. *c* under potential pulsing conditions, the effective thickness is smaller in a partially swollen film compared with that of the film in *b*, which swells over the entire volume. *d* three-dimensional enlargement of the partially swollen film under a 1-s positive potential pulse (Δ*E* = 0.6, 0.5, or 0.4 V). The caffeine-imprinted cavities in the swollen outer part (marked by *d*) are opened up for caffeine binding; however, those embedded inside the nonswollen part near the Au electrode are not. *PBS* phosphate-buffered saline (Adapted from [94])

A simple mechanism of reversible modulation of the number of caffeine cavities imprinted in an MIPPy film under electric stimuli was postulated [94]. This number was adjusted to optimize the detectability of the chemosensor using this film with respect to a given test solution. In equilibrium (Fig. 5, state a), the MIPPy film structure was essentially closed. That is, the caffeine-imprinted cavities remained inaccessible for the caffeine molecules in solution. Hence, the MIPPy chemosensor in this state was unable to detect any caffeine at all. If a positive potential (0.6 V vs. SCE) was applied, however, solvated anions of the supporting electrolyte penetrated the MIPPy film for compensation of the positive charge generated in it. This penetration caused swelling of the polymer (Fig. 5, state b). An open porous structure thus formed allowed the caffeine molecules in solution to enter the MIPPy film and occupy the imprinted cavities. In this swollen state, practically all imprinted cavities became available for caffeine binding and, hence, for caffeine detection. That way, the MIPPy chemosensor was ready for determination of caffeine in a wider (10–40 μM) concentration range than that (3–10 μM) at low positive potential, i.e., 0.4 V. However, the film swelled only partially (Fig. 5, states c, d) when a series of well-defined positive potential 1-s pulses of Δ*E* = 0.5 V or Δ*E* = 0.4 V amplitude were applied. Then, the supporting electrolyte ions could enter only the polymer bulk adjacent to the electrolyte. Therefore, the outer part of the film, i.e., that near the MIPPy–electrolyte interface, swelled more than the inner part, i.e., that near the Au electrode surface (Fig. 5, state d). Caffeine in solution freely accessed the imprinted cavities in the swollen part of MIPPy, thus defining the effective thickness, *d*, of the film. In contrast, the inner part of the film remained in a closed form impeding penetration of caffeine from the solution. In this state, caffeine binding was limited to the cavities of the swollen outer part of the MIPPy film, resulting in a *d* value lower than that of the total film thickness. The number of accessible cavities was, therefore, much lower than that generated in the whole MIPPy film. Thus, the film was virtually turned into an ultrathin construct, which enabled caffeine determination at a low limit of detection (LOD) of 3 μM (Table 1).

Paracetamol analgesic [95], sulfamethoxazole antibiotic [96], and ascorbic acid (AA; vitamin C) [97] templated MIPPy films were potentiodynamically deposited on pencil-graphite electrodes (PGEs) to fabricate differential pulse voltammetry (DPV) miniature chemosensors for determination of the respective medicine analyte. The pH, monomer and template concentrations, and the number of potential cycles applied for electropolymerization were optimized (Table 1).

Moreover, L-Asp-templated thin MIOPPy films were galvanostatically deposited on Au-coated quartz resonators [98]. Mass changes of the MIOPPy films during electropolymerization and overoxidation were determined by PM. The deposition by electropolymerization from a weakly acidic (pH = 6.0) solution of Py and an L-Asp salt lead to nonenantioselective MIOPPy films. In contrast, electropolymerization of an alkaline Py solution containing L-Asp resulted in an adherent, smooth, and homogeneous L-Asp-templated MIOPPy film, which after overoxidation was enantioselective at pH = 1.6. The L-Asp template was removed by galvanostatic overoxidation at constant current density of 25 μA cm^-2^ in phosphate buffer (pH = 7.0). Selectivity with respect to L-Asp and D-Asp of the respective MIP film was relatively high, L/D = 20 (Table 1).

For enantioselective determination of D-phenylalanine (D-Phe) and L-Phe, a few micrometer long and approximately 100 nm in diameter MIPPy nanowires were prepared by galvanostatic electropolymerization [99]. In this polymerization, an enantiomeric D-camphorsulfonic acid (D-CSA) or L-CSA chiral resolving agent acted as both the dopant and the surrogate template. Owing to low solubility of Py in water and surface activity of CSA, Py and CSA spontaneously formed micelles. In addition, CSA behaved as a protecting agent preventing radial overgrowth of the MIPPy nanowires. Therefore, these nanowires could just be grown. Both the electrochemical impedance spectroscopy (EIS) and the circular dichroism spectra demonstrated enantioselective interactions between the de-doped PPy nanowires, imprinted with the CSA enantiomers, and the D-Phe or L-Phe analyte (Table 1).

In investigations of the mechanism of the potentiostatic deposition of MIPPy films in the presence of glutamate, the electropolymerization conditions, including concentrations and the concentration ratio of the template and functional monomer as well as the solution pH, were optimized [100]. It appeared that the mechanism was unique, being different from that of the analogous mechanism of deposition of the film of MIPPy templated with glutamate in an alkaline solution (pH = 9.0). Thicker films were deposited from a neutral solution rather than acidic or basic solutions and the glutamate excess enhanced deposition of the film. The glutamate strongly interacted with Py in solution, apparently forming a stable complex.

A PM chemosensor was constructed for the potential-dependent uptake and release of L-glutamate in neutral solutions under CV (−0.8 and 0.6 V vs. Ag/AgCl) conditions [101]. The device was composed of an Au electrode coated by potentiostatic electropolymerization with an (L-glutamate)-templated MIOPPy film. The (L-glutamate)-template was then removed by galvanostatic overoxidation at constant current density of 25 μA cm^-2^ in phosphate buffer (pH = 7.0). The enantioselectivity of the MIOPPy film with respect to L-glutamate over D-glutamate in neutral solutions, validated by PM, was L/D = 3.5.

A voltammetric chemosensor using a potentiodynamically grown MIPPy film was devised for determination of a 2,4-dichlorophenoxyacetic acid (DCPA) systemic pesticide [102]. During the electropolymerization, DCPA molecules were embedded in the MIPPy matrix by both hydrogen bonds and electrostatic interactions. The DCPA template was then removed by potentiostatic overoxidation at 1.30 V in 0.2 M Na_2_HPO_4_. This chemosensor was satisfactorily selective with respect to plausible interfering substances, including methyl parathion, methamidophos, chlorpyrifos (CPF), AA, salicylic acid, and 3,4-dihydrobenzoic acid. However, the calibration curve was linear in a relatively narrow concentration range (Table 1).

L-Tryptophan (l-Trp) and D-Trp templated MIOPPy films were used as recognition units of PM chemosensors for enantioselective determination of these amino acids [103]. Apparently, L-Trp was nearly twice as deeply inserted in the MIPPy film as D-Trp. The Trp template was removed by overoxidation of the MIPPy film in 0.1 M NaOH at 1.0 V vs. SCE. The analytical performance of the MIOPPy films was examined by PM using an electrochemical quartz crystal microbalance (Table 1). As expected, L-Trp rather than D-Trp was embedded in the MIPPy film closer to the Au film electrode of the quartz resonator.

A zearalenone mycotoxin was templated in an MIPPy film potentiostatically deposited on an Au electrode [104]. Then, the zearalenone template was removed from the film by consecutive washing with acetonitrile (ACN), methanol, and chloroform. The MIPPy–surface plasmon resonance (SPR) chemosensor fabricated that way exhibited an appreciably broad linear concentration range of response to zearalenone (Table 1). The selectivity of the chemosensor with respect to zearalenone and the structurally related interfering analogues α-zearalenol, β-zearalenol, zearalanone, and α-zearalanone was 0.15, 0.21, 0.25, and 0.27, respectively.

In another study, a trichloroacetic acid TCAA-templated non-(cross-linked) MIPPy thin film was galvanostatically deposited on a quartz resonator [105]. Then, the TCAA template was extracted with distilled water. The TCAA-templated MIPPy film, integrated with PM transduction, revealed a TCAA-dependent linear frequency shift, whereas the TCAA-dependent current response of this film combined with CV transduction was linear over a much wider concentration range (Table 1).

A new functional monomer, ethylenediamine tetra-*N*-(3-pyrrole-1-yl)propylacetamide, was used for potentiostatic preparation of MIP films on carbon disc electrodes for DPV determination of selected metal cations, such as Cu^2+^ and Hg^2+^ [106]. In this monomer, four polymerizable Py moieties were covalently inserted in the skeleton of ethylenediaminetetraacetic acid (EDTA), which, additionally, was used both to improve the rigidity and the three-dimensional structure of the film. Electropolymerization of this functional monomer was inefficient in the presence of the Cu^2+^ template owing to the strong oxidizing properties of the Cu^2+^–ACN complex. This complex chemically oxidized the functional monomer and caused rapid degradation of the polymerization solution. Therefore, Zn^2+^ was used as the surrogate template to prepare the MIP for Cu^2+^. The imprinted Hg^2+^ and Zn^2+^ ions were extracted from their respective MIP films by soaking the films in 0.01 M sodium dithiocarbamate and 0.01 M EDTA, respectively.

An MIPPy film of the SPR chemosensor for an ochratoxin (OTA) food-contaminating mycotoxin was prepared by potentiostatic electropolymerization of Py on an SPR chip from an ethanol–water (1:9, *v*:*v*) solution of OTA [107]. The film growth was manifested in situ by an increase in the SPR angle. The MIPPy film was regenerated by pulsed elution of the OTA template with 1% acetic acid in a methanol–water (1:9, *v*:*v*) solution (Table 1).

A recent very interesting area of MIP analytical research involves preparation of chemosensors for determination of complete biological cells and even viruses. A Py functional monomer appeared quite suitable for imprinting of these large objects [108, 109]. Toward that, conducting MIP films for determination of *Bacillus subtilis* endospores were synthesized [108]. The films were prepared by adsorbing the endospores on (PPy-film)-coated GCEs followed by depositing a poly(3-methylthiophene) film by potentiostatic electropolymerization of 3-methylthiophene. Next, the endospore template was released by soaking the resulting bilayer construct in dimethyl sulfoxide. Binding of endospores to the imprinted film was detected via EIS by monitoring changes in susceptance (*Y*) with 0.5 M MnCl_2_ (pH 3.0) used as the supporting electrolyte. Here, Mn^2+^ also acted as an ion bridging to the affinity site on the MIP film and the endospore surface. Detection of the absorbed spore was more sensitive after endospore germination via CV changes of the film charge. Unfortunately, regeneration of the MIP films was not possible without losing their activity.

In another study, a chronoamperometric MIPPy chemosensor for bovine leukemia virus glycoprotein, gp51, was devised [109]. The gp51 template was extracted from the MIPPy film with 1 M H_2_SO_4_. Binding of gp51 to the MIP film was detected with pulse chronoamperometry (Table 1). However, the reusability of the MIPPy film was unsatisfactory owing to the necessity of using highly acidic solvent for extraction.

These determinations of whole cells or viruses [108, 109] employed a more flexible noncovalent approach to imprinting in aqueous solutions. MIP syntheses in aqueous solutions may lead to higher selectivity and stronger binding of water-soluble target molecules. However, improvement in regeneration protocols appeared to be necessary because of vital effects of these protocols on binding and selectivity with respect to the viruses.

The above examples indicate that Py has most often been chosen as the functional monomer for molecular imprinting. An MIPPy film can be positively charged, thus allowing imprinting of anionic template molecules. The resulting film subsequently exhibits high selectivity. Because of this effect, chemosensors for enantiomeric amino acids have successfully been devised (Table 1).

#### Aminophenylboronic acid based functional monomers

Understanding the intermolecular interactions between binding sites of analytes and recognition sites of imprinted cavities that allow molecular recognition is essential for proper MIP design. As a sensing material, a polymer of APBA can reversibly mediate recognition of different biomacromolecules. In this polymer, the boronic acid moiety binds compounds containing vicinal diol moieties with high affinity through reversible esterification. This covalent binding allows the construction of chemosensors, e.g., for carbohydrates and nucleotides. With that in mind, high interest has emerged in studying the interactions between boronic acids and different diol-containing compounds.

For instance, a carbohydrate-templated molecularly imprinted PAPBA (MIPAPBA) film can reversibly recognize carbohydrates [110]. For that, a new approach to electrochemical synthesis of a saccharide-templated MIP of APBA has been developed (Table 2). Accordingly, a (D-fructose)-templated MIP film was prepared by potentiodynamic electropolymerization of APBA in the presence of D-fructose and F^-^ under either slightly acidic (pH = 5.0) or neutral conditions. The role of F^-^ was to disrupt intermolecular B–N interactions between the APBA monomers. In the resulting self-doped MIP, an anionic boronic ester complex was formed between APBA and D-fructose. For template removal, the (MIP-film)-coated electrode was soaked overnight in phosphate-buffered saline (PBS) solution (pH = 7.4). The potentiometric response of the MIP film was selective for the D-fructose analyte over the D-glucose interferent, suggesting the usefulness of the approach developed for manipulation of the selectivity of the boronic acid reactions of complex formation (Table 2).

**Table 2:** Analytical parameters of MIP chemosensors using phenylboronic acid derivatives as the functional monomers

Template/analyte	Transduction/electrode	Electropolymerization conditions	Solution for MIP preparation	Solution of analyte determination	Linear dynamic concentration range	LOD	*K*_MIP-temp_ (M^-1^)	Reference
d-Fructose	Potentiometry/GCE	Potentiodynamic −0.1 to 1.0 V vs. Ag/AgCl	0.5 M phosphate buffer, 40 mM NaF (pH 5.0-7.4)	0.5 M phosphate buffer (pH 7.4)	-	-	-	[110]
Nucleotide, monosaccharides	PM/Au	Potentiodynamic 0.1 to −1.4 V vs. Ag/AgCl	0.2 M ZnCl_2_	-	Up to 17 mM (nucleotide)	-	-	[111]
	EIS/Au	Potentiodynamic 0.1 to −1.4 V vs. Ag/AgCl	0.2 M ZnCl_2_	0.05 M HEPES buffer (pH 7.4)	-	-	-	
Glucose	Potentiometry/ISFET	Chemical oxidation	0.01 M PBS, 0.14 M NaCl (pH 7.0)	Phosphate buffer solution	1 μM to 0.8 mM	0.8 μM	-	
AMP					30 μM to 5 mM	15 μM	-	
GMP					40 μM to 5 mM	15 μM	-	
CMP					2 μM to 0.5 mM	0.8 μM	-	
UMP					Up to 5 mM	10 μM	-	
Lysozyme, Cytochrome	CV/Pt	Potentiodynamic −0.2 to 0.7 V vs. Ag/AgCl	0.5 M PBS, 120 mM NaF (pH 5.5)	0.5 M PBS (pH 5.5)	Up to 37.5 mg L^-1^	-	-	[112]
					Up to 60 mg L^-1^	-	-	
T-2 toxin	SPR/Au	Potentiodynamic 0 to 1.1 V vs. Ag/AgCl	0.05 M NaNO_3_	Phosphate buffer (pH 7.5)	2.1–33.6 fM	0.1 fM	7.8 × 10^13^	[113]
Neomycin	SPR/Au	Potentiodynamic −0.35 to 0.85 V vs. Ag/AgCl	0.1 M HEPES buffer (pH9.2)	0.1 M HEPES buffer (pH 9.2)	Up to 1 μM	2.0 pM	-	[114]
Kanamycin						1.0 pM		
Streptomycin						0.2 pM		

*AMP* adenosine monophosphate, *GMP* guanosine monophosphate, *CMP* cytosine monophosphate, *UMP* uridine monophosphate, *EIS* electrochemical impedance spectroscopy, *ISFET* ion-selective field-effect transistor, *HEPES* 4-(2-hydroxyethyl)-1-piperazineethanesulfonic acid

Nucleotide-imprinted acrylamide–acrylamidephenylboronic acid copolymer films were deposited by potentiodynamic electropolymerization on an Au electrode of a quartz resonator for PM determinations or on an Au wire electrode for faradaic EIS determinations, or they were deposited by free-radical polymerization on the gate of an ion-selective field-effect transistor [111]. For imprinting, the film-coated transducer was soaked in a solution of the respective nucleotide after every few potential cycles. The nucleotide template was extracted from the resulting MIP film with 1% NH_4_OH (Table 2).

In another investigation involving a voltammetric MIP chemosensor for lysozyme and cytochrome *c*, a screen-printed Pt electrode was coated with three layers of different polymer films [112]. That is, on the first (innermost), PPy layer, deposited by potentiodynamic electropolymerization, a second (intermediate) thin layer of PAPBA was potentiodynamically deposited. The third (outermost) layer was prepared by potentiodynamic electropolymerization of APBA either in the presence of the protein template to form an MIPAPBA film or in the absence of the template to serve as a respective control nonimprinted PAPBA film. Next, the templating protein was extracted with 3% acetic acid which was 0.1% in Tween 20. The anodic CV peak current of the redox moiety of the polymer decreased owing to bonding of the nonconducting protein analyte to the polymer surface. This decrease was used to measure the extent of analyte binding (Table 2). The detectability of the MIPAPBA films was markedly improved by depositing them on supporting layers of PPy. PPy is a fully conjugated polymer, which permits chain-to-chain “hopping” of electrons. Therefore, it overcomes the conductivity restriction imposed by the intermediate –NH– groups linking two APBA moieties.

For SPR determination of T-2 toxin (T-2), an MIPAPBA film was in situ deposited on an SPR chip by potentiodynamic electropolymerization of APBA, in the presence of T-2 [113]. Next, the template was removed by washing first with 80% methanol and then with PBS (pH = 7.4) which was 0.05% in Tween 20. The values of thermodynamic functions, such as the Gibbs free-energy change (Δ*G*), enthalpy change (Δ*H*), and entropy change (Δ*S*), indicated that the interaction between T-2 and the MIPAPBA film was spontaneous, endothermic, and entropy-driven. Superior binding affinity of the MIPAPBA film for T-2 was confirmed by a high value of the stability constant of the MIP–T-2 complex, *K*_MIP–T-2_ = 7.8 × 10^13^ M^-1^ (Table 2). The MIPAPBA film was selective for ricin, curcin, microcystine-LR, and abrin interferents.

**Fig. 6:**
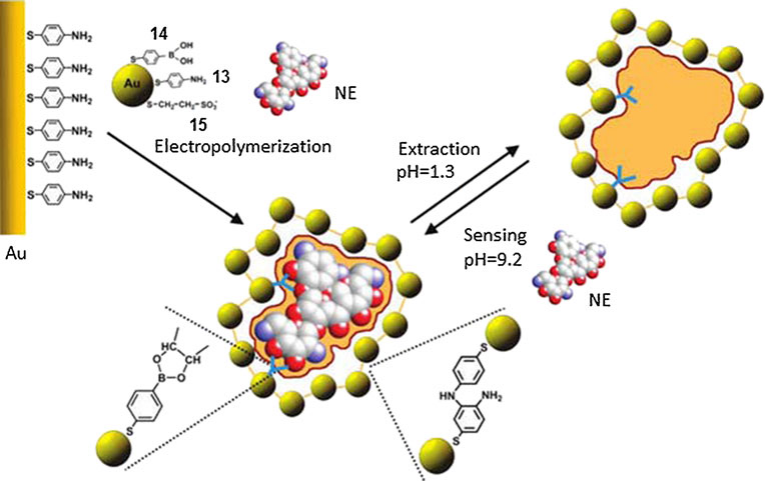
Mechanism of imprinting of molecular cavities featuring sites for recognition of antibiotics, for example, neomycin (NE), through electropolymerization of a 4-thioaniline **13** cross-linked Au nanoparticle composite with 4-mercaptophenylboronic acid **14** as a functional monomer on an Au electrode surface. Mercaptoethanesulfonic acid **15** prevented coagulation of Au nanoparticles. (Adapted from [114])

Electropolymerizable 4-thioaniline **13** modified Au nanoparticles (AuNPs) (Fig. 6), coated with a boronic acid functionalized thiol ligand, 4-mercaptophenylboronic acid **14** (Fig. 6), were used for preparation of MIP films for SPR determination of a series of antibiotics bearing vicinal diol groups. They included neomycin, kanamycin, and streptomycin [114]. Mercaptoethanesulfonic acid **15** (Fig. 6) additionally modifying AuNPs stabilized them, thus preventing coagulation. The MIP film was potentiodynamically grown on an Au SPR chip modified with a self-assembled monolayer (SAM) of 4-thioaniline. The imprinted antibiotic template was then removed by acid hydrolysis. The detectability and selectivity of these MIP films for the imprinted antibiotics was high (Table 2).

The studies described above concluded that the boronic acid based functional monomers can generate complexes of MIPs and analytes with high stability constants [113], comparable to those of enzyme- or antibody-based biorecognition systems. However, in most studies, these stability constants were not reported (Table 2). Moreover, boronic acid based MIPs were successfully integrated, as recognition units, with different transducers (Table 2). The linear concentration range was wide and detectability was high for most of them (Table 2).

#### Thiophene-based functional monomers

PTh and its derivatives are among the most frequently used ECPs [115] because of their intrinsically high electrical conductivity (~10^6^ S m^-1^), chemical and mechanical stability, and processability in both their doped and undoped states [116]. Like PPy and PANI, PTh can be either oxidatively or reductively doped in a suitable solvent [117]. The former is possible because the presence of sulfur in the PTh structure enables its reduction and, therefore, p-doping [115]. PTh films prepared by anodic electropolymerization have been widely used for sensing applications (Table 3) [40, 118–121].

**Table 3:** Analytical parameters of MIP chemosensors using thiophene derivatives as the functional monomers

Template/analyte	Transduction/electrode	Electropolymerization conditions	Solution for MIP preparation	Solution for analyte determination	Linear dynamic concentration range	LOD	*K*_MIP-temp_ (M^-1^)	Reference
Histamine	PM/Pt	Potentiodynamic 0 to +1.5 V vs. Ag/AgCl	0.1 M (TBA)ClO_4_/ACN	0.5 M HEPES (pH 7.5)	10–100 mM	5 nM	57	[37]
Dopamine	PM/Pt	Potentiodynamic 0 to +1.5 V vs. Ag/AgCl	0.1 M (TBA)ClO_4_/ACN	0.2 M PBS (pH 7.0)	0.1–1 mM	10 nM	4.46 × 10^7^	[38]
Adenine	PM/Pt	Potentiodynamic 0 to +1.5 V vs. Ag/AgCl	0.1 M (TBA)ClO_4_/ACN (pH = 6.0)	ACN/water (1:1, v:v)	0.5–7 mM	5 nM	1.8 × 10^5^	[39]
Melamine	PM/Pt	Potentiodynamic 0 to +1.5 V vs. Ag/AgCl	Ionic liquid/ACN (1:1,v:v) (pH 3.0)	0.1 M HCl	5 nM to 1 mM	5 nM	1.813 × 10^4^	[40]
Morphine	Chronoamperometry/ITO	Potentiostatic +1.2 V vs. Ag/AgCl	0.1 M LiClO_4_/ACN	0.1 M KCl (pH 5.3)	Up to 1 mM	0.2 mM	-	[124]
Morphine	Chronoamperometry/Pt	Potentiodynamic	0.1 M LiClO_4_/ACN	0.1 M KCl	0.01–0.2 mM	0.3 μM	-	[125]
Atrazine	Voltammetry/Pt	Potentiostatic +1.45 V vs. Pt (10 s)	0.1 M TBATFMS/CH_2_Cl_2_	0.1 M TBATFMS/CH_2_Cl_2_	Up to 15 mM	0.1 μM	-	[126]
Avidin	Fluorescence/Au	Potentiodynamic −0.2 to +1.1 V vs. Ag/AgCl	0.025 M NaPSS	0.05 M PBS	100 ng mL^-1^ to 1 mg mL^-1^	-	2.5 × 10^6^	[128]
Naproxen, paracetamol, theophylline	SPR/Au	Potentiodynamic 0 to +0.8 V vs. Ag/AgCl	0.1 M (TBA)PF_6_	0.1 M PBS	10–50 μM (theophylline)	-	-	[130]
Theophylline	SPR, PM/Pt	Potentiodynamic 0 to +0.8 V vs. Ag/AgCl	0.1 M (TBA)PF_6_/ACN	PBS	10–50 μM	3.36 μM	-	[131]
Folic acid	PM/Pt	Potentiodynamic 0 to +1.1 V vs. Ag/AgCl	0.1 M (TBA)PF_6_/ACN	ACN/water (1:9, v:v)	Up to 100 μM	15.4 μM	2.6 × 10^4^	[132]

*ITO* indium tin oxide, *TBATFMS* tetrabutylammonium trifluoromethane sulfonate

For close integration of the MIP recognition unit with the transducer surface, an intermediate ECP layer was utilized to immobilize conventional MIPs in chronoamperometric chemosensors [122, 123]. For instance, morphine-templated MIP particles, prepared by free-radical polymerization of a methacrylic acid functional monomer and a trimethylolpropane trimethacrylate cross-linker, were immobilized on an indium tin oxide electrode with the use of a PEDOT layer [122]. For that, EDOT was first deposited by potentiostatic electropolymerization at 1.2 V vs. Ag/AgCl. The morphine template was extracted from the MIP particles with methanol. Moreover, EDOT alone was used as a functional monomer for imprinting of morphine (Table 3) [124].

A microfluidic system for determination of morphine was constructed by combining MIP recognition and chronoamperometric transduction [125]. Toward that, a molecularly imprinted PEDOT MIPEDOT-modified electrode was prepared by potentiodynamic electropolymerization of EDOT onto a Pt microelectrode in an ACN solution of EDOT and morphine. The resulting MIPEDOT-modified microelectrode was integrated in a dedicated microfluidic system. In fact, two sets of microelectrodes were used for improving the selectivity for morphine. The first set played the role of a preseparator for adsorption of AA. The second one served to determine morphine (Table 3). The morphine template was extracted from the MIP film with methanol.

In one study, 3-thiophene acetic acid (TAA) was used as a functional monomer to imprint an atrazine herbicide owing to its ability to hydrogen bond to this template [126, 127]. The hydrophilicity of an EDOT cross-linker counterbalanced the hydrophobicity of the TAA monomer [126]. The atrazine template was removed from the MIP matrix with a solution of methanol and acetic acid (7:3, v:v) protic solvents. Atrazine binding to MIP was quantified by the electrooxidation charge determined by integration of the area under CV curves (Table 3).

Although preparation of MIPs for determination of small molecules is now straightforward, imprinting of large structures, such as proteins or entire cells, remains a challenge. The major difficulty in imprinting of these large objects consists in their restricted mobility within highly cross-linked polymer networks and, hence, the low efficiency of template removal and analyte binding. Therefore, surface imprinting of these objects seems to be the most promising way to overcome these difficulties. Because the imprinted sites in such an imprinting are situated at or close to the surface of MIPs, they are easily accessible to target protein molecules or cells.

In that direction, surface-imprinted PEDOT microrods, doped with poly(styrene sulfonate) (PSS), were used for selective fluorescent determination of an avidin protein under potentiostatic square-wave pulse sequence conditions [128]. For this determination, (8-μm)-diameter precisely sized cylindrical micropores of a track-etched polycarbonate membrane (PCM) were used as sacrificial microreactors for synthesis of these microrods. The hydrophobic nature of the PCM membrane allowed straightforward fixing of the avidin target onto the pore walls of the PCM by physical adsorption. Then, this membrane was placed on the Au electrode surface to potentiostatically grow in its micropores microrods of PEDOT doped with PSS. Subsequent dissolution of the PCM sacrificial material with chloroform resulted in formation of microrods vertically confined to the Au electrode with the complementary imprint of the avidin target on their surfaces. Functional groups of the PEDOT–PSS material were capable of generating hydrogen bonds as well as electrostatic and π–π interactions with the protein template. Notably, formation of the latter interactions was more plausible because avidin is rich in Trp (Table 3).

Recently, another procedure was developed for electropolymerization of EDOT around living cells [129]. That is, an MIPEDOT film was deposited by galvanostatic electropolymerization around neurons cultured on an Au/Pd electrode surface. The embedded neurons remained viable for at least 120 h after the electropolymerization. The electropolymerization was impeded in regions where electrochemically active neural cells adhered well to the electrode surface. Subsequent cell removal with a trypsin–EDTA solution generated a neural-cell-templated biomimetic conducting MIPEDOT film featuring cell-shaped cavities that were attractive to these cells. Surprisingly, the electrical properties of the electrode coated with the neural-cell-templated MIPEDOT film were significantly improved compared with those of the bare electrode. This improvement could be due to the electrically active nature of the neural cells, which might facilitate the charge transfer between the electrode and the MIPEDOT film [129].

**Fig. 7:**
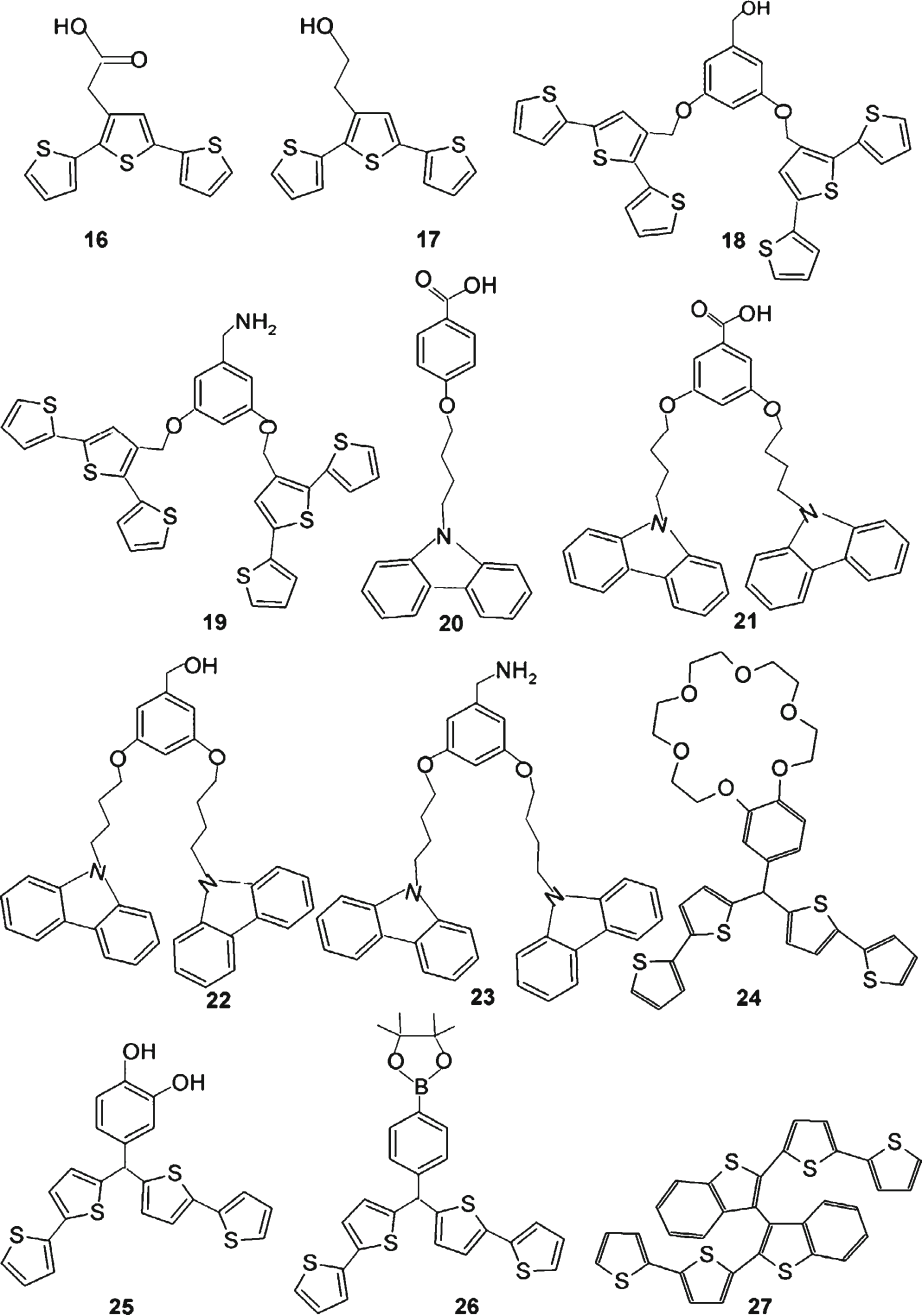
Structural formulas of derivatives of thiophene (**16**–**19**, **24**–**27**) and carbazole (**20**–**23**) used as functional (**16**–**26**) and cross-linking (**27**) monomers for molecular imprinting

Derivatized functional monomers (Fig. 7) have triggered the emergence of a new generation of ECP-based MIPs, in which electronic and electrochemical properties of the conjugated backbone are combined with molecular recognition. This new class of advanced molecular materials allowed the development of facile approaches to prepare tailor-made, highly selective, and robust ultrathin MIP films for determination of pharmaceuticals, such as theophylline, paracetamol, naproxen [130, 131], and folic acid [132], as well as biogenic amines, such as histamine [37], dopamine (DA) [38], adenine [39], and melamine [40] (Table 3).

An MIP film prepared by potentiodynamic electropolymerization of terthiophene derivatives **16**–**19** (Fig. 7) was used as a recognition unit for an SPR chemosensor. The SPR detection signal of bifunctional monomers bearing –COOH and –OH functional groups **18** was higher than that of monofunctional monomers **16**, **17**. This effect was due to a higher number template–monomer complexes formed per unit volume of the MIP film for the former. The bifunctional –NH_2_ carbazole **23** and terthiophene-based monomers **16**–**19** more weakly bound analytes owing to weaker hydrogen bonding by the –NH_2_ group than by the –COOH or –OH group. In some instances, a constant potential of 0.4 V vs. Ag/AgCl was applied to the film for template extraction with ACN, thus facilitating the release of the template because of film swelling [130, 131]. For a folic acid template, the MIP film was prepared by potentiodynamic electropolymerization of a bisterthiophene dendron functional monomer from an ACN solution of tetrabutylammonium hexafluorophosphate [132]. The folic acid template was subsequently removed with a mixed methanol–acetic acid (9:1, *v*:*v*) solvent solution. Unfortunately, the cross-selectivity of the folic acid imprinted polymer against interferents, such as pteroic acid, caffeine, and theophylline, was low. This indicated significant cross-interactivity of the MIP cavities and interferent molecules. Moreover, other drugs, such as paracetamol and naproxen, were imprinted using the same functional and cross-linking monomers [130]. The selectivity and detectability of the chemosensor with respect to these drugs for the interferents 3-aminophenol, acetanilide, and 3-aminobenzoic acid were appreciable (Table 3).

For imprinting of more selective molecular cavities in an MIP film, different derivatives of bithiophene (Fig. 7) have been used as functional monomers. For instance, derivatives including benzo-[18-crown-6]-bis(2,2′-bithien-5-yl)methane **24**, *meso-*(3,4-dihydroxyphenyl)-bis(2,2′-bithien-5-yl)methane **25**, and [4-(5,5-dimethyl-1,3,2-dioxaborinane-2-yl)-phenyl]-bis(2,2′-bithien-5-yl)methane **26** were used for imprinting of histamine [37], DA [38], adenine [39], and melamine (Table 3) [40]. In this imprinting, protonated primary amine groups of the templates were complexed by the benzo-18-crown-6 moiety of **24**, the diol groups of DA formed hydrogen bonds with the 3,4-dihydroxyphenyl substituent of **25**, and the imine nitrogen atom of the imidazole or purine moiety of histamine and adenine, respectively, was coordinated by the boron atom of the dioxaborinane substituent of **26**. The detectability and selectivity of the resulting MIP films were largely increased by cross-linking the functional monomers with the electroactive {[2,2′-bis(2,2′-bithiophene-5-yl)]-3,3′-bithianaphthene} **27** cross-linking monomer and by the presence of the trihexyl(tetradecyl)phosphonium tris(pentafluoroethyl)-trifluorophosphate porogenic ionic liquid in the prepolymerization solution [40].

However, a problem arises in the case of imprinting by electropolymerization of a redox template if this template is electroactive under the conditions of the electropolymerization of the functional monomer used. Then, products of the template electrode reaction may be imprinted instead of the genuine template itself. Moreover, these products may adsorb and, consequently, foul the electrode surface. Therefore, the electrode reaction of such a template should be eliminated. Toward that, at least two imprinting procedures are available. In one, an electroinactive analogue is used as the template instead, as exemplified in free-radical polymerization of methacrylic acid monomer [133–136]. In another, a barrier underlayer of the ECP is first deposited, which prevents electrochemical transformation of the template, on the one hand, and allows electrodeposition of the MIP film, on the other [37–39].

Some functional and cross-linking monomers can electropolymerize in aqueous solutions, whereas others require nonaqueous media. Several studies have confirmed that preparation of MIPPy in aqueous solutions is possible (Table 1). In contrast, PTh is synthesized in an organic solvent (e.g., ACN) solution (Table 3). The scarce water solubility of Th and its oxidation potential being higher than that of water are two main reasons why Th does not electropolymerize in water solutions. Additionally, the thienyl radical cation R (Fig. 3) can react with water molecules, which hampers the Th electropolymerization. However, for a real (mostly biological) world application of MIPs, it is desired to electropolymerize electroactive functional monomers in aqueous media. For that, one can envision the use of acidic solutions, anionic micelles, or supramolecular hosts (such as cyclodextrins) as the media for electropolymerization of Th-based water-insoluble functional monomers.

#### Porphyrin-based functional monomers

Stable and electrode-adherent metalloporphyrin MIP films were prepared by electropolymerization of different porphyrin functional monomers. These monomers offer at least two kinds of recognition. One is a metal ion center, which can bind a heteroatom of a template through donation of electrons from this atom to the unfilled orbitals of the outer coordination sphere of the metal ion. The other is a derivatized peripheral part of the porphyrin, which can form hydrogen bonds or participate in electrostatic interactions with the template.

**Fig. 8:**
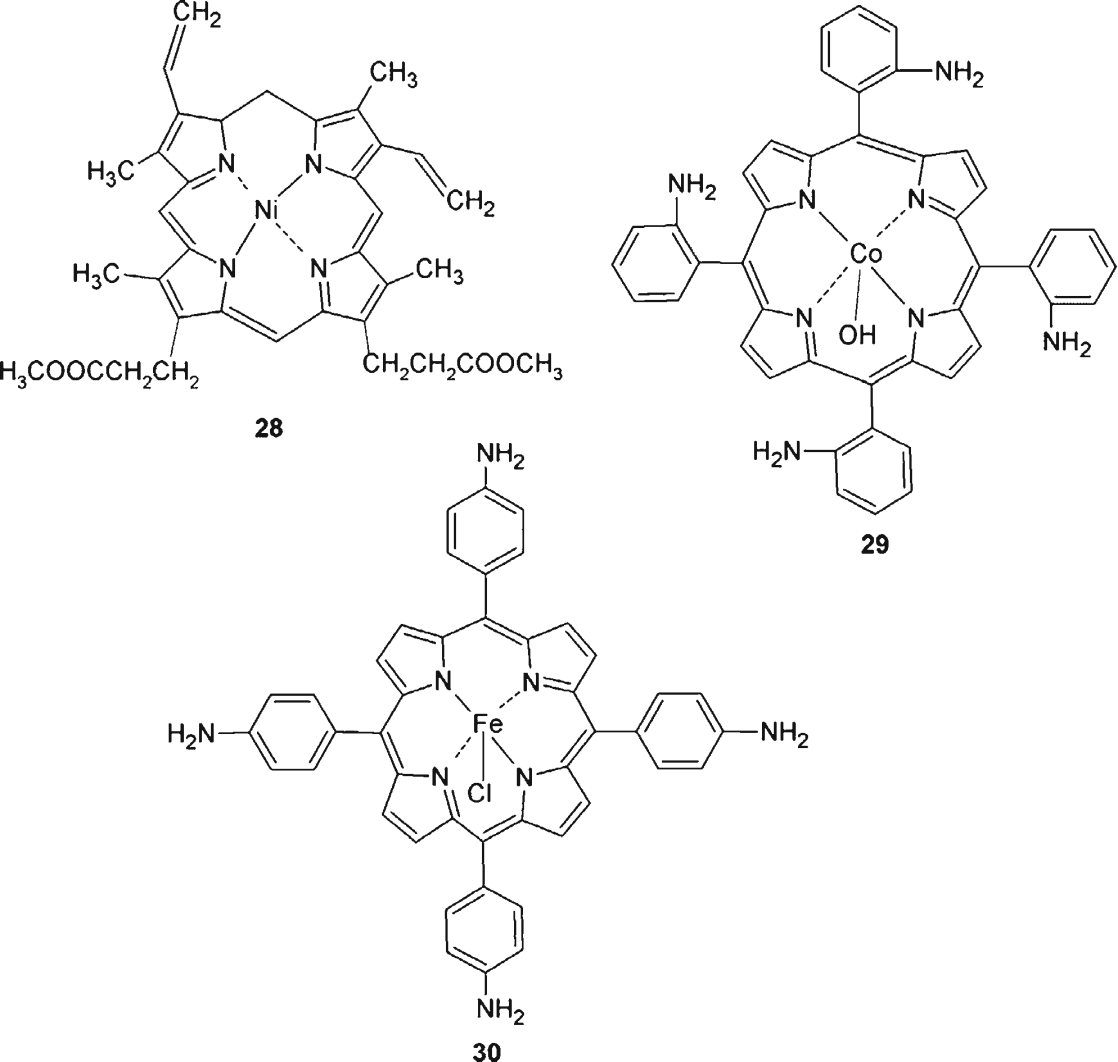
Structural formulas of the porphyrin functional monomers nickel protoporphyrin IX dimethyl ester **28**, cobalt(III) tetrakis(2-aminophenyl)porphyrin hydroxide **29**, and iron(III) tetrakis(4-aminophenyl)porphyrin chloride **30**

In that direction, a voltammetric MIP chemosensor for nitrobenzene was prepared. For that, a GCE was coated with an MIP film by potentiodynamic electropolymerization of a nickel protoporphyrin IX dimethyl ester **28** functional monomer (Fig. 8) in the presence of the nitrobenzene template [137]. Although nitrobenzene detectability was poor, the MIP film discriminated nitrobenzene from the interferents 2-nitrotoluene and 3-nitroaniline (Table 4).

**Table 4:** Analytical parameters of MIP chemosensors using porphyrins as the functional monomers

Template/analyte	Transduction/electrode	Electropolymerization conditions	Solution for MIP preparation	Solution of analyte determination	Linear dynamic concentration range	LOD	Reference
Nitrobenzene	CV/GCE	Potentiodynamic 0.5 to 1.5 V vs. Ag/AgCl	Dichloromethane	0.1 M phosphate buffer (pH 7.0)	Up to 1 M	0.01 M	[137]
DCPB	Chronoamperometry/Pt	Potentiodynamic −0.1 to 1.0 V vs. Pt	0. 1 M (TBA)PF_6_/ACN	0. 1 M TBAPF_6_/ACN	200 μM to 2 mM	-	[139]
Dopamine	SWV/Pt	Potentiodynamic −0.2 to 1.0 V vs. Ag/AgCl	0.1 M Na_2_SO_4_/H_2_SO_4_ (pH 1.0)	0.1 M acetate buffer	1–100 μM	0.4 μM	[140]

*DCPB* 2,4-dichlorophenoxybutyric acid, *SWV* square wave voltammetry

A cobalt porphyrin was used for fabrication of a chronoamperometric and voltammetric MIP chemosensor for determination of 4-(2,4-dichlorophenoxy)butyric acid, an organohalide [138, 139]. For that, an MIP film was potentiodynamically deposited onto a Pt electrode by using cobalt(III) tetrakis(2-aminophenyl)porphyrin **29** (Fig. 8) as the functional monomer. The template was then extracted by extensive washing of the film with ACN followed by washing with methanol (Table 4).

Oxidation of peripheral aminophenyl substituents of an iron(III) tetra(4-aminophenyl)porphyrin functional monomer **30** (Fig. 8) favored electrochemical synthesis of a very stable porphyrin polymer with the phenazine-like structures binding together porphyrin macrocycles. Because of this advantage, a novel square wave voltammetry (SWV) chemosensor selective for DA was devised (Table 4) [140]. For that purpose, iron(III) tetra(4-aminophenyl)porphyrin polymer was deposited by potentiodynamic electropolymerization on a carbon fiber microelectrode. The template was then removed from the film with 0.1 M phosphate buffer (pH = 8.0).

#### Other functional monomers electropolymerizing to form conducting polymers

Polyazine MIP films appeared useful for fabrication of chemosensors [141]. For instance, a methylene green functional monomer was deposited by potentiodynamic electropolymerization onto a GCE to fabricate a molecularly imprinted poly(methylene green) (MIPMG) recognition unit of a voltammetric chemosensor for theophylline (Table 5). In the course of this electropolymerization, a cation radical was formed and then dimerized to yield an –NH– bridge between two monomer molecules. The template was removed with 1.0 M Na_2_SO_4_. Surprisingly, the voltammetric response to the theophylline analyte of the nonimprinted poly(methylene green) coated electrode was higher than that of the MIPMG-coated electrode. That was because binding of theophylline to molecular cavities of MIPMG decreased the effective diffusion coefficient of theophylline in the MIP film, resulting in the lower current response. The MIPMG-film-coated GCE discriminated the theophylline analyte from the caffeine interferent (Table 5).

**Table 5:** Analytical parameters of MIP chemosensors using other functional monomers

Functional monomer	Template/analyte	Transduction/electrode	Electropolymerization conditions	Solution for MIP preparation	Solution of analyte determination	Linear dynamic concentration range	LOD	Reference
Methylene green	Theophylline	CV/GCE	Potentiodynamic −0.3 to 1.3 V vs. SCE	100 mM NaNO_3_,	-	3–75 μM	-	[141]
				10 mM NaB_4_O_7_				
Aminobenzoic acid	Melamine	DPV/GCE	Potentiodynamic −0.8 to 1.0 V vs. SCE	0.2 M Na_2_SO_4_	0.2 M Na_2_SO_4_	4–450 μM	0.36 μM	[142]
MBI	Pyrene	SWV/Au	Potentiodynamic −0.6 to 1.3 V vs. Ag/AgCl	Ethanol/water (7:3,v:v) (pH 10)	100 mM NaClO_4_, 50% ethanol (pH 7.4)	Up to 0.35 μM	-	[186]
MBI	Cholesterol	Capacitance/Au	Potentiodynamic −0.6 to 1.3 V vs. Ag/AgCl	0.1 M NaClO_4_ (pH9.5)	10 mM PBS (pH 7.4)	5–30 μM	0.42 μM	[187]
MBI	Cholesterol	DPV/Au	Potentiodynamic −0.6 to 1.3 V vs. SCE	Ethanol (pH 9.5)	Ethanol/water (1:1, v:v)	Up to 20 μM	0.7 μM	[188]
MBI	Fenvalerate	Capacitance/Au	Potentiodynamic 0 to 1.9 V vs. SCE	Ethanol (pH 7.5)	5 mM PBS, 140 mM NaCI (pH = 7.5)	Up to 5 μg mL^-1^	0.36 μg mL^-1^	[189]

*MBI* 2-mercaptobenzimidazol, *DPV* differential pulse voltammetry

Recently, 4-aminobenzoic acid (AMA) was also used as a functional monomer of an MIP for melamine [142]. The recognition and selectivity with respect to melamine was facilitated by formation of hydrogen bonds between melamine and AMA moieties of molecularly imprinted poly(4-aminobenzoic acid). The melamine template was then extracted from the MIP film with an ACN–water (1:1, *v*:*v*) solution. In indirect melamine quantization, the decrease in the DPV peak current of the K_3_Fe(CN)_6_ redox probe was proportional to the melamine concentration in solution (Table 5).

Although ECPs are conducting, it is unlikely that any redox analyte can exchange charge at the surface of such a polymer coating an electrode. In most cases, recognition of an analyte by molecular cavities, imprinted for its determination, is necessary. This recognition is electrochemically manifested by the use of the K_4_Fe(CN)_6_ probe [37–40]. If the analyte molecules occupy molecular cavities, the current due to the electrode reaction of the probe is negligibly small; however, this current is high if the analyte molecules are removed from these cavities. This gate effect proves that emptying the cavities enables diffusion of the probe through the ECP film to the electrode.

### Electrosynthesized nonconducting polymers in molecular imprinting

Several electroactive functional monomers, including PD, Ph, and thiophenol (Fig. 1b), electropolymerize forming nonconducting polymers. On the basis of this property, these monomers were most often used to fabricate either PM or capacity MIP chemosensors.

#### 1,2-Phenylenediamine-based functional monomers

The PD functional monomer is quite suitable for molecular imprinting because it grows to form compact (rigid) polymer films, which offer hydrophilic, hydrophobic, basic, and other recognition sites (Table 6) [143–145]. In addition, these films can be made thin and continuous, a feature required to afford a short response time of a chemosensor [143].

**Table 6:** Analytical parameters of MIP chemosensors using 1,2-phenylendiamine as the functional monomer

Template/analyte	Transduction/electrode	Electropolymerization conditions	Solution for MIP preparation	Solution for analyte determination	Linear dynamic concentration range	LOD	*K*_MIP-temp_ (M^-1^)	Reference
Glucose	PM/Pt	Potentiodynamic 0 to 0.8 V vs. Ag/AgCl	Acetate buffer (pH 5.2)	Acetate buffer (pH 5.2)	Up to 20 mM	-	2.1 × 10^2^	[146]
Glucose	Capacitance/Au	Potentiodynamic 0 to 0.8 V vs. Ag/AgCl	0.01 M acetate buffer (pH 5.18)	0.1 M NaCl, 10 mM Tris buffer (pH 7.14)	0.1–20 mM	0.05 mM	-	[147]
Atropine	PM/Au	Potentiodynamic −0.1 to 0.8 V vs. SCE	0.1 M H_2_ SO_4_	0.1 M H_2_ SO_4_	8.0 μM to 4.0 mM	2.0 μM	-	[148]
Phenylalanine	PM/Au	Potentiodynamic 0.1 to 0.8 V vs. SCE	0.1 M acetate buffer (pH = 6.0)	0.1 M acetate buffer (pH = 6.0)	2–20 mM	0.5 mM	1.98 × 10^2^	[149]
Sorbitol	PM/Au	Potentiodynamic 0.1 to 0.8 V vs. SCE	Acetate buffer (pH 5.2)	0.05 M PBS (pH 6.8)	1–15 mM	1 mM	11	[150]
Glutathione	Capacitance/Au	Potentiodynamic 0 to 0.8 V vs. SCE	0.02 M PBS (pH 6.98)	0.1 M KCl, 0.02 M PBS (pH6.98)	0.025–0.30 mM	1.25 μM	-	[151]
DCPA	SWV/PGE	Potentiodynamic +1.0 to −1.0 V vs. Ag/AgCl	4% DMSO	1 M KCl	-	-	-	[152]
DCPA	Chronoamperometry/Au	Potentiodynamic +1.0 to −1.0 V vs. Ag/AgCl	2% DMSO	1 M KCl	Up to 8 μg mL^-1^	-	-	[153]
Paracetamol	Voltammetry/carbon	Potentiodynamic −0.1 to +1.35 V vs. Ag/AgCl	0.1 M H_2_SO_4_	0.1 M PBS (pH 7.0)	6.5 μM to 2.0 mM	1.5 μM	-	[154]
2-Methyl-4,6-dinitrophenol	Voltammetry/carbon	Potentiodynamic −0.1 to +1.35 V vs. Ag/AgCl	0.2 M H_2_SO_4_ methanol (1:1, v:v)	0.04 M BR buffer (pH3) methanol (9:1, v:v)	0.8 μM to 100 μM	0.2 μM	-	[155]
Metamitron	Voltammetry/carbon	Potentiodynamic −0.1 to +1.35 V vs. Ag/AgCl	0.1 M H_2_SO_4_	0.04 M BR buffer	1–100 μM	0.27 μM	-	[156]
DHP	Voltammetry, Impedimetry/GCE	Potentiodynamic 0 to 0.8 V vs. SCE	1/15 M PBS (pH 6.98)	1/15 M PBS (pH 6.98)	50 nM to 50 μM	10 nM	-	[157]
Dimethoate	Voltammetry/Au	Potentiodynamic 0 to 0.8 V vs. SCE	Acetate buffer (pH 5.2)	0.1 M PBS (pH = 7.0)	1.0–1,000 ng mL^-1^	0.5 ng mL^-1^	-	[158]
					1.0–50 μg mL^-1^			
Theophylline	Voltammetry/GCE	Potentiodynamic 0 to 0.8 V vs. SCE	0.1 M acetate buffer (pH5.2)	-	0.4–15 μM	0.1 μM	-	[159]
					0.24–3.4 mM			
Triclosan	Chronoamperometry/GCE	Potentiodynamic 0 to 0.8 V vs. SCE	Acetate buffer (pH 5.2)	Acetate buffer (pH 5.2)	0.2–3.0 μM	0.08 μM	-	[160]
Dopamine	Voltammetry/Au	Potentiodynamic 0 to 0.8 V vs. SCE	0.1 M acetate buffer (pH6.5)	0.1 M PBS (pH 7.0)	0.5–40 μM	0.13 μM	-	[161]
Oxytetracycline	Voltammetry/Au	Potentiodynamic 0 to 0.8 V vs. Ag/AgCl	0.1 M acetate buffer (pH5.2)	0.1 M PBS (pH 7.2)	Up to 1 μM	0.64 nM	-	[162]
Oxytetracycline	Voltammetry/Au	Potentiodynamic 0 to 0.8 V vs. Ag/AgCl	0.1 M acetate buffer (pH5.2)	0.1 M PBS (pH 7.2)	Up to 0.4 μM	33 nM	5.34 × 10^3^	[163]
DMP	SAW/Au	Potentiodynamic 0 to 0.8 V vs. Ag/AgCl	-	N_2_ gas	0.19–19.6 mg mL^-1^	0.1 mg mL^-1^	-	[164]

*DCPA* 2,4-dichlorophenoxyacetic acid, *DHP O*,*O*-dimethyl-*α*-hydroxyphenyl phosphonate, *DMP* dimethylmethylphosphonate, *SAW* surface acoustic wave, *DMSO* dimethyl sulfoxide

Glucose is an extremely important therapeutic target for monitoring. For that, a glucose-templated molecularly imprinted poly(1,2-phenylenediamine) (MIPPD) film was prepared by potentiodynamic electropolymerization and applied as a recognition unit of a PM biomimetic chemosensor. This polymer was the first example of electrochemically synthesized MIP film for a neutral template [146]. Imprinting of a neutral template is difficult with the Py functional monomer because of the necessity of overoxidation of the resulting MIPPy film. Therefore, the PD monomer was used instead as the functional monomer of choice for glucose imprinting under improved selectivity and sensitivity conditions (Table 6). After potentiodynamic electropolymerization, the template was removed with distilled water. The Scatchard analysis of the derived calibration plot showed two types of molecular cavities characterized by two different complex stability constants. They were *K*’_MIP-glu_ = 29 and *K*”_MIP-glu_ = 210 M^-1^ for the high and low glucose concentrations, respectively. Presumably, glucose was strongly bound in the imprinted cavities at lower concentrations, whereas it resided also within the MIPPD network and was more weakly bound at higher concentrations. In another study, a capacity glucose MIP chemosensor was fabricated [147]. MIP films often suffer from pinholes or defects because a film preferentially grows on top of the polymer just being deposited rather than on the bare electrode substrate. Therefore, structural defects in the MIPPD film of this glucose chemosensor were blocked by a SAM of dodecane-1-thiol. Distilled water was then used to remove the template from the MIPPD film. Glucose binding by the resulting MIPPD film was recognized by the capacity decrease (Table 6).

Several MIP chemosensors with PM transduction were devised by electropolymerization of PD [148, 149]. For that, ANI and PD were potentiodynamically copolymerized on surface of the Au electrode of a quartz resonator for imprinting of an atropine sulfate alkaloid. The atropine template was subsequently washed away from the MIP film with distilled water. The relationship between the resonant frequency shift (Δ*f*) and log *C* was linear in a wide concentration range (Table 6) [148]. Moreover, PD alone was used as a functional monomer for imprinting of DL-Phe. The Phe template was removed from the film with 0.01 M acetic acid followed by 0.01 M acetate buffer (pH = 6.0) (Table 6) [149].

Accumulation of sorbitol in patient tissues may cause diabetic complications. Toward PM sorbitol determination, a sorbitol-templated MIPPD film was deposited by potentiodynamic electropolymerization of PD onto an Au electrode of a quartz resonator [150]. The templating sorbitol was removed with distilled water (Table 6).

A capacity chemosensor for a glutathione tripeptide antioxidant was prepared by potentiodynamic electropolymerization of PD, in the presence of the glutathione template, on an Au electrode precoated with a SAM of 2-mercaptoethanesulfonate [151]. Pinhole defects of the resulting MIPPD film were patched with a dodecane-1-thiol SAM, and this improved the performance of this chemosensor. That was because this SAM greatly influenced the insulating property of the film. The glutathione template was removed from the MIP film by basic hydrolysis. The dielectric property of the film and the analytical parameters of the chemosensor were characterized by DPV (Table 6).

Resorcinol and PD were potentiodynamically coelectropolymerized in the presence of a templating dye, such as fluorescein, rhodamine [152], or 2,4-dichlorophenoxyacetic acid (DCPA) [153], to deposit MIPPD films on pencil-graphite electrodes (PGEs) for chronoamperometric determination of the dyes (Table 6). After electropolymerization, the MIPPD films were washed from the templates with distilled water followed by washing with methanol until no dye was fluorescently detected [152]. The cathodic current responses at −1.0 V vs. Ag/AgCl of the resulting chemosensors proved higher affinity for templating than for nontemplating dyes [153] (Table 6).

PD and ANI functional monomers were used for preparation of MIP-based SWV chemical microsensors for a paracetamol pain reliever [154], 2-methyl-4,6-dinitrophenol pesticide [155], and metamitron herbicide (Table 6) [156]. To prepare MIP films with the highest density of imprinted molecular cavities, different electropolymerization conditions were examined. The paracetamol [154], 2-methyl-4,6-dinitrophenol [155], and metamitron [156] templates were extracted from the resulting respective MIP films with 0.1 M phosphate buffer (pH = 7.0) water–methanol (6:4, *v*:*v*) solution, and 0.5 M ammonia buffer (pH = 10.0), respectively.

An MIP film for capacity chemosensing of an *O*,*O*-dimethyl-α-hydroxylphenyl phosphonate (DHP) pesticide was prepared by electropolymerization of PD in the presence of DHP on a GCE [157]. The DHP template was then extracted with ethanol. In the ethanol solution, the capacity of the MIPPD-film-coated GCE was higher than that of the bare electrode. Presumably, either the effective area of the MIP film was higher or the distance of the electrolyte to the electrode was lower for the former (Table 6).

To improve detectability of MIP-based chemosensors, metal nanoparticles (NPs), such as Ag [158] and Au [159], were deposited over MIP films. This combination resulted in a large specific surface area and high biocompatibility. This procedure was used for devising voltammetric MIP chemosensors for a dimethoate insecticide [158] and theophylline, a respiratory disease drug [159]. For that, first, an MIPPD film was deposited by potentiodynamic electropolymerization of PD in the presence of the template. Subsequently, potentiostatic deposition of the metal NPs on top of the deposited MIPPD film resulted in a bilayer of NPs and the MIPPD films. In both cases [158, 159], templates were extracted with ethanol. The NPs provided conducting pathways for transfer of electrons, thus promoting electrode reaction of the K_3_Fe(CN)_6_ probe. The analytes were determined by measuring the dependence of the peak current of the probe on the analyte concentration. This current linearly decreased with the analyte concentration increase in two ranges (Table 6).

Moreover, a voltammetric MIPPD chemosensor for determination of a triclosan antibacterial and antifungal agent was prepared by potentiodynamic electropolymerization of PD in the presence of the triclosan template on a GCE [160]. Then, the template was extracted with 0.1 M NaOH. Unfortunately, the chemosensor linearly responded to triclosan over a rather narrow concentration range (Table 6).

A sensitive and selective voltammetric chemosensor for DA was prepared by potentiodynamic coelectropolymerization of PD and resorcinol in the presence of the DA template [161]. Next, this template was removed from the MIP film with 0.1 M PBS (pH = 7.4). This film was selective for DA because the negatively charged functional groups of the MIP strongly attracted electrostatically the DA cations and repelled anions, such as the AA and uric acid residues. Moreover, functional groups, such as −NH_2_ and −OH, of the functional monomer formed strong hydrogen bonds with diol groups of the DA template (Table 6).

**Fig. 9:**
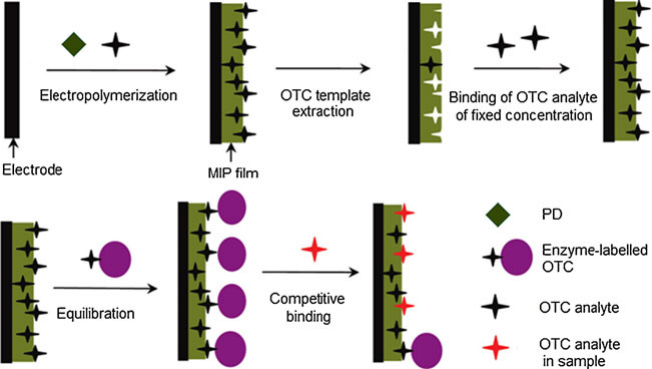
Procedure for preparation of the molecularly imprinted polymer chemosensor for surface plasmon resonance determination of oxytetracycline (OTC) using phenylenediamine (PD) as the functional monomer. (Adapted from [163])

An oxytetracycline (OTC) antibiotic for treatment of bacterial infections was indirectly determined by using voltammetric MIP artificial immunosensors [162, 163]. A competition reaction between the enzyme-labeled OTC and pristine OTC served for this immunosensing. Two enzymes were used for OTC labeling, i.e., glucose oxidase [162] and horseradish peroxidase [163], in order to construct two different artificial immunosensors. PD was potentiodynamically electropolymerized in the presence of OTC to form MIPPD films (Fig. 9). Then, the OTC template was removed with 20% HNO_3_. The analytical performance of these immunosensors was characterized by EIS, DPV, and CV (Table 6) [162].

A surface acoustic wave (SAW) chemosensor was prepared by coating a 300-MHz SAW transducer with an MIPPD film used as the recognition unit for gas-phase sensing of a dimethylmethylphosphonate (DMP) flame retardant (Table 6) [164]. Sarin acid, a structural analogue of DMP, was used as a template for preparation of this MIPPD film. However, the reason for selecting sarin acid over DMP was not explained. The MIPPD film was deposited on the Au electrode of the SAW device by potentiodynamic electropolymerization of PD in the presence of the sarin acid surrogate template. The template was extracted with distilled water from the resulting MIPPD film. A SAW delay line on the X-propagating quartz (ST-X) resonator with low insertion loss and single-mode selection capability was fabricated as the oscillator element. To structure the SAW device, an electrode-width control single-phase unidirectional transducer configuration and comb transducer was used to minimize the insertion loss and to accomplish the single-mode selection, respectively. Prior to fabrication, simulation of modes was coupled to predict the performance of this chemosensor.

MIP films with a well-defined structure for capacity recognition of GA enantiomers were grown on Au electrodes by potentiodynamic copolymerization of PD and DA in the presence of L-GA or D-GA (Table 6) [165]. Next, the L-GA or D-GA template was extracted from the resulting MIP films with 50 mM HCl followed by distilled water. The films were characterized by CV, capacity, atomic force microscopy, and X-ray photoelectron spectroscopy measurements. Copolymerization of DA with PD as well as the compactness and the smoothness of the MIP films were confirmed by X-ray photoelectron spectroscopy and atomic force microscopy. The structure and recognition ability of the films were strongly dependent on the MIP composition. The selectivity for L-GA and D-GA of the respective MIP film was L/D = 24, and D/L = 15. Under the same preparation conditions, the D-GA imprinted film grew thicker than the L-GA imprinted film. For this thicker film, recognition of D-GA was worse owing to higher D-GA nonspecific adsorption. Therefore, the enantiomeric selectivity for L-GA exceeded that for D-GA.

The linear concentration range of most MIP-based chemosensors using PD functional monomers is broad, and the LOD values are mutually comparable, sometimes extending to the nanomolar concentration range (Table 6). In contrast, MIP chemosensors prepared using acrylic monomers suffer from much higher LODs [166, 167]. Moreover, MIPs using PD functional monomers were successfully integrated with different transducers to fabricate MIPPD chemosensors.

#### Phenol-based functional monomers

Ph-based MIP films are widely used as recognition units in chemical sensors because of their straightforward preparation. Moreover, these films can interact with many different analytes through *π*–*π* stacking.

A capacity MIP chemosensor was devised for determination of Phe [168]. Toward that, first, an underlayer of mercaptophenol was self-assembled on an Au electrode. Then, Ph was potentiodynamically electropolymerized in the presence of the Phe template to result in a molecularly imprinted polyphenol (MIPPh) film. Pinhole defects in this film were filled with a 4-mercaptophenol SAM. Next, the template and nonbound 4-mercaptophenol were washed away with PBS (pH = 7.5). However, the chemosensor fabrication procedure was not optimized with respect to temporal stability and reversibility, as both these parameters were very poor. Nevertheless, small effects of interferents and high reproducibility allowed this chemosensor to be considered as a single-use device (Table 7).

**Table 7:** Analytical parameters of electrochemical MIP chemosensors using phenol as the functional monomer

Template/analyte	Transduction/electrode	Electropolymerization conditions	Solution for MIP preparation	Solution for analyte determination	Linear dynamic concentration range	LOD	*K*_MIP-temp_ (M^-1^)	Reference
Phenylalanine	Capacitance/Au	Potentiodynamic 0 to 0.8 V vs. SCE	0.05 M NaHPO_4_ (pH 7.5)	-	0.5–8 mg mL^-1^	-	-	[168]
Ferritin	Voltammetry/carbon	Potentiodynamic 0 to 0.9 V vs. Ag/AgCl	PBS (pH 7.4)	PBS (pH 7.4)	Up to 0.1 μg L^-1^	10 pg L^-1^	0.019 (pg/ L)^-1^	[169]
d-Glucose	Chronoamperometry/Au	Potentiodynamic 0 to 0.35 V vs. SCE	0.1 M phosphate buffer (pH 10.3)	0.05 M HEPES buffer (pH 7.2)	Up to 2.0 mM	-	170	[170]
d-Mannose					Up to 1.0 mM	-	870	
MV^2+^	Voltammetry/Au	Potentiodynamic 0 to 0.6 V vs. SCE	0.05 M PBS (pH 7.4)	0.05 M PBS (pH 7.4)	Up to 10 mM	-	3.45 × 10^3^ M^-1^	[171]
DCPA	PM/Au	Potentiodynamic −0.2 to +1.0 V vs. SCE	0.1 M NaClO_4_, 0.4 M HClO_4_	0.05 M HClO_4_	40 μM to 2.0 mM	10 μM	-	[172]
NIC	Voltammetry/Au	Potentiodynamic −0.2 to +1.0 V vs. SCE	NaClO_4_	-	0.4–33 μM	0.2 μM	-	[173]
Tegafur	Capacitance/PM/Au	Potentiodynamic 0 to 0.8 V vs. SCE	1/15 M PBS (pH 6.98)	1/15 M PBS (pH 6.98)	Up to 1 mM	-	6.25 × 10^4^ M^-1^	[174]
Dopamine	Voltammetry/Au	Potentiodynamic −0.2 to +1.2 V vs. Ag/AgCl	0.1 M NaClO_4_ (pH 5.5)	0.2 M NaCl, 0.1 M PBS (pH 7.2)	0.02 - 0.25 μM	1.98 nM	-	[175]
Theophylline	Capacitance/Au	Potentiodynamic 0.2 to +0.8 V vs. Ag/AgCl	0.05 M borate buffer (pH 9.2)	0.05 M borate buffer (pH 9.2)	Up to 15 μM	1 μM	-	[176]

*MV* ^*2+*^ methyl viologen, *NIC* nicotine

As discussed above, proteins are highly challenging targets of molecular imprinting. This is because the sheer bulk of MIPs can noncovalently entrap protein, whereas the diverse functionality of MIPs can covalently immobilize them, particularly when the MIP is prepared as a bulk monolith. In recent pioneer work, a protein-templated MIPPh film was potentiodynamically deposited on the tips of arrayed carbon nanotubes to fabricate an impedimetric chemosensor [169]. A potential of 0.30 V vs. Ag/AgCl was first applied for 30 s to the tips of the carbon nanotubes to attract the protein template. Subsequent extraction with a 5% acetic acid and 5% DS solution of the templating protein from the accessible surface of the MIPPh nanocoatings was manifested by the decrease in electric impedance. Using EIS, the chemosensor determined proteins, such as human ferritin and the E7 oncoprotein derived from human papillomavirus, with appreciable detectability. That is, the protein analyte was recognized by an increase in the impedance due to the relatively low conductivity of the protein-loaded MIPPh film (Table 7).

A polyphenol-functionalized imprinted boronic acid served for enantioselective chronoamperometric determination of monosaccharides [170]. Accordingly, MIP films for selective determination of D-glucose and D-mannose were deposited by potentiodynamic coelectropolymerization of Ph and 3-hydroxyphenylboronic acid in the presence of the monosaccharide. The monosaccharide template was then extracted with 0.1 M HCl. The monosaccharides were determined by competitive chronoamperometric measurements employing ferrocene-modified monosaccharides as redox probes (Table 7).

An MIPPh film for recognition of *N,N*’-dimethyl-4,4′-bipyridinium (methyl viologen, MV^2+^) was prepared by potentiodynamic electropolymerization of Ph in the presence of MV^2+^ [171]. Then, the template was washed away with 50 mM phosphate buffer (pH = 7.4). The electropolymerized 1,4-dialkoxybenzene unit exhibited *π*-donor properties manifested by formation of a (*π* donor)–(*π* acceptor) complex with MV^2+^ during electropolymerization, which led to the MIPPh film. The association to and dissociation from the MIPPh imprinted sites of MV^2+^ was examined by CV (Table 7).

2-Aminophenol (APh) was potentiodynamically electropolymerized in the presence of DCPA and the properties of the resulting molecularly imprinted poly(2-aminophenol) film were investigated by PM [172]. The DCPA template was then extracted with doubly distilled water. During the electropolymerization, changes in the resonant frequency (Δ*f*), dynamic resistance (Δ*R*), and static capacitance (Δ*C*_0_) were simultaneously measured. A distinct drop in Δ*f* and Δ*R* indicated efficient electropolymerization of APh. Although the selectivity of the chemosensor was not superior, the chemosensor readily determined DCPA (Table 7).

Moreover, APh was potentiodynamically electropolymerized in a weak acidic solution (pH = 3 to 8) of a nicotine template and this resulted in a molecularly imprinted poly(2-aminophenol) film for nicotine determination using DPV [173]. H_2_SO_4_ (0.5 M) subsequently removed the nicotine template from the film. The selectivity and detectability of the chemosensor with respect to nicotine in the presence of other alkaloids, such as palmatine and berberine hydrochloride, were appreciable. Unfortunately, they were inferior in the presence of the atropine sulfate interferent (Table 7).

In addition, several other MIPPh chemosensors for electrochemical determination of an analyte, such as a tegafur prodrug of 5-flurouracil [174], DA [175], and theophylline [176], were fabricated.

#### Thiophenol-based monomers

Several appealing features of SAMs of alkyl thiols make them useful for many analytical applications. Strong binding of these SAMs to Au and other solid surfaces provides effective ways for molecular imprinting. However, the main drawback of this imprinting was the low stability of the resulting non-cross-linked imprinted film. Recognition sites of this film can easily be destroyed after template removal. The selectivity and kinetics of association were strongly affected by the length of the alkanethiol chains. For instance, it was difficult to prepare recognition sites if these chains were too short, on the one hand, and they could block these sites with their flexible chains if these chains were too long, on the other [177]. In contrast, electropolymerized SAMs appeared superior for depositing a sensing film directly on the transducer surface. For instance, molecular imprinting was combined with electropolymerization of a SAM of 2-AThPh, resulting in an MIP film for determination of nitrobenzene [178] and a metolcarb pesticide [179].

Syntheses of MIP matrices assembled on metal NPs, such as AuNPs, demonstrated significant advantages in determination of the explosive 2,4,6-trinitrotoluene (TNT) [180, 181]. Accordingly, AuNPs were modified with electropolymerizable thioaniline functional monomer, then deposited by electropolymerization onto the Au electrode in the presence of the TNT template or a dummy template. In effect, affinity for the thioaniline groups and for the electrogenerated bisaniline bridges was induced. On this basis, voltammetric [180] and SPR [181] chemosensors were devised (Table 8). For the systems of low MIP coverage with the analytes or association of a low molecular weight analyte with the MIP film, the dielectric changes generated at the Au surface were too small to allow detectable SPR spectral changes. Conjugation of the MIP film with AuNPs served to amplify the SPR recognition signal of the TNT analyte. This increase resulted both from formation of a large number of *π*-donor sites on the electrode surface and from three-dimensional conductivity of the AuNP matrix.

**Table 8:** Analytical parameters of MIP chemosensors using thiophenol as the functional monomer

Template/analyte	Transduction/electrode	Electropolymerization conditions	Solution for MIP preparation	Solution of analyte determination	Linear dynamic concentration range	LOD	*K*_MIP-temp_ (M^-1^)	Reference
Nitrobenzene	Chronoamperometry/Au	Potentiodynamic −0.4 to 1.0 V vs. SCE	CH_2_Cl_2_	PBS (pH 7.0)	0.5–4.5 mM	-	-	[178]
Metolcarb	Chronoamperometry/Au	Potentiodynamic −0.2 to 1.4 V vs. SCE	5 mM (TBA)ClO_4_, 10 mM HCl	0.2 M KNO_3_	50–350 nM	13.4 nM	-	[179]
TNT	LSV/Au	Potentiodynamic −0.35 to 0.5 V vs. SCE	0.1 M phosphate buffer (pH 7.4)	0.1 M phosphate buffer (pH 7.4)	-	46 pg mL^-1^	2.6 × 10^4^	[180]
TNT	SPR/Au	Potentiodynamic −0.35 to 0.8 V vs. Ag	0.1 M HEPES buffer (pH 7.2)	0.1 M HEPES buffer (pH 7.2)	20–100 fM	10 fM	6.4 × 10^12^	[181]
Picric acid, MV^2+^	SPR/Au	Potentiodynamic −0.35 to 0.8 V vs. Ag	0.1 M HEPES buffer (pH 7.2)	0.1 M HEPES buffer (pH 7.2)	50 fM to 2 pM	-	3.9 × 10^12^	[182]
					1–100 nM	-	9.0 × 10^7^	
Chlorpyrifos	CV/GCE	Potentiodynamic −0.2 to 0.6 V vs. Ag/AgCl	0.05 M PBS (pH 6.86), 0.1 M KCl	0.05 M PBS (pH 6.86), 0.1 M KCl	0.5–10 μM	0.3 μM	-	[183]
Tolazoline	CV/Au	Potentiodynamic −0.4 to 1.2 V vs. SCE	Acetate buffer (pH 5.2)	PBS (pH 6.8), 0.1 M NaCl	0.05–5 μg mL^-1^	16 ng mL^-1^	-	[184]
					5.0–240 μg mL^-1^			

*TNT* 2,4,6-trinitrotoluene, *LSV* linear sweep voltammetry

Recently, composites of 4-aminothiophenol (4-AThPh) and bisaniline-cross-linked AuNPs were used for electrochemically triggered accumulation and release of different analytes by an SPR chemosensor of an MIP (Table 8) [182]. For that, a film of 4-AThPh was deposited by potentiodynamic electropolymerization to functionalize an Au electrode. In this bisaniline-cross-linked AuNP matrix of an MIP, potential-controlled reduction and oxidation of the bisaniline cross-linking units governed the (*π* donor)–(*π* acceptor) interactions between the bisaniline bridging units and the *π*-acceptor analytes, such as picric acid and MV^2+^. Application of a negative potential of −0.30 V vs. Ag to the imprinted matrix resulted in an SPR signal increase, consistent with absorption of MV^2+^ by the composite. Application of a positive potential of 0.40 V resulted in a decrease of the SPR signal to the baseline value, indicating MV^2+^ release.

A surface molecular self-assembly strategy was adopted for molecular imprinting of a CPF pesticide to fabricate a voltammetric MIP chemosensor [183]. Toward that, a 4-AThPh monolayer was first self-assembled on a GCE surface precoated with AuNPs. That way, the CPF template was anchored to the 4-ATPh monolayer through hydrogen bonds between the amine group of 4-AThPh and the nitrogen or oxygen atom of CPF. An MIP-film-based recognition unit was then fabricated on the Au electrode assembled that way by subsequent potentiodynamic electropolymerization of the surface film in a mixed solution of additional amounts of the 4-AThPh monomer and the CPF template (Table 8).

An MIP chemosensor was devised for voltammetric determination of tolazoline (TL), an α-adrenergic blocker [184, 185]. For that, an electrode functionalized with a SAM of 2-ATPh was first prepared by immersing a bare Au electrode for 20 h in an acetate buffer (pH = 5.2) solution of 2-ATPh and the TL template [184]. After that, the TL-templated SAM of 2-ATPh was potentiodynamically electropolymerized. Then, AuNPs were attracted to the resulting MIP-film-coated electrode to enhance the sensitivity and to amplify the voltammetric response of the chemosensor. Next, the TL template was removed with 0.2 M HCl. The CV peaks were proportional to the TL concentration in two ranges (Table 8). The first range corresponded to filling of imprinted cavities of the MIP by the TL molecules and the second to TL absorption by the MIP network.

#### Other functional monomers electropolymerizing to form nonconducting polymers

As mentioned already, thiol chemistry appeared feasible to fabricate MIP-film-based chemosensors. Toward that, SAM-coated Au electrodes were devised for SWV determination of pyrene [186]. Actually, two different procedures were developed to prepare MIP films on Au electrodes. In one procedure, 2-mercaptobenzimidazole (MBI) was potentiodynamically deposited on an Au electrode in the presence or absence of the pyrene template to produce an MIP and nonimprinted polymer film, respectively. Next, this template was removed from the former film by multiple washing with toluene, ethanol, and then water to allow determination of pyrene. In the other approach, an *N*-(1-pyrenyl)maleimide derivative of pyrene was covalently bound to 1,3-propanethiol, which was subsequently self-assembled onto an Au electrode surface. Resorcinol was then potentiodynamically electropolymerized onto this modified electrode. Finally, the thiolated pyrene template was removed by voltammetric stripping between −0.2 to +1.2 V vs. Ag/AgCl. For each electrode, binding of pyrene to the MIP-coated electrode was indirectly detected with SWV through the pyrene-film-dependent access of a hexacyanoferrate redox probe to the electrode surface (Table 5).

Determination of cholesterol in food and blood is important in dietetics as well as in clinical analysis and diagnosis. Toward this end, capacity MIP chemosensors for cholesterol were fabricated (Table 5) [187, 188]. For that, an MIP film was prepared by potentiodynamic electropolymerization of a SAM of MBI on an Au electrode in the presence of the cholesterol template. The electrode surface left uncoated with this film was then saturated with a SAM of dodecane-1-thiol to make the surface layer compact [187]. The cholesterol template was removed from the film with an ethanol–water (4:1, *v*:*v*) solution of 0.2 M NaOH (pH = 13).

Furthermore, MBI was used as a functional monomer for imprinting of fenvalerate, a synthetic pyrethroid insecticide [189]. For that, the MIP film was deposited on an Au electrode by potentiodynamic electropolymerization in the presence of the fenvalerate template. Templating fenvalerate was then extracted with an ethanol–water (4:1, *v*:*v*) solution of 0.2 M NaOH (pH = 13). Unfortunately, the capacity chemosensor prepared that way lost 50% of its original activity after 10 days of storage (Table 5).

All of the above examples illustrate the use of electroactive functional monomers for preparation of recognition units of chemosensors. The resulting conducting or nonconducting MIPs have certain advantages and disadvantages. For instance, if a nonconducting MIP film is deposited by electropolymerization, its thickness is self-limited. This is because its deposition will be stopped if a thickness is reached at which the polymer insulates the underlying conducting electrode surface. In contrast, deposition of ECPs by electropolymerization should proceed indefinitely. The polymer thickness can be then controlled by the conditions of the deposition. The conductivity of the polymer also determines the choice of the method of signal transduction. For instance, impedance changes can more easily be detected with a nonconducting polymer.

## Conclusions

As evidenced with numerous studies, a lack of effective uniform procedures for MIP immobilization on a transducer surface is still one of the most important hindrances for the application of acrylic- or vinylic-based MIPs for chemosensing. In contrast, deposition of thin MIP films directly on surfaces of conducting transducers using electroactive functional monomers effectively overcomes this drawback.

MIPs prepared by electropolymerization of electroactive functional monomers reveal several advantages over MIPs prepared by free-radical polymerization combined with drop-coating or spin-coating of a solution of a monomer or preformed polymer. Particularly, the film thickness can be conveniently controlled during electropolymerization by the electrochemical parameters of the polymerization and deposition. Furthermore, application of suitable solvents and supporting electrolytes can tune the viscoelastic properties and porosity of the MIP films.

Recent achievements in molecular imprinting involving ECPs indicate that the possibility of direct chemosensing of MIP electrochemical devices based on ECPs is enormous. MIP films using ECP matrices facilitate the charge transport between the electrode substrate and the analyte occupying molecular cavities of the MIP. In addition, there is a possibility of increasing the conductivity of these films by integrating them with conducting NPs such as AuNPs or carbon nanotubes to form composite MIP matrices. Moreover, synthesis and use of derivatized electroactive functional monomers helped to fabricate more selective ECP-based MIPs. These derivatized electroactive functional monomers contain additional functional groups, which form stable complexes with templates during a preorganization step. Stabilization of these complexes is further enhanced by *π*-delocalized bonds in polymer chains. Therefore, these electroactive functional monomers have successfully been used in imprinting of different target analytes ranging from amino acids to pesticides, drugs, and proteins.

Electropolymerization resulting in nonconducting MIP films appeared preferably useful for fabrication of PM and capacity chemosensors. For capacity-based MIP chemosensors, PD and Ph are very suitable as functional monomers. After electropolymerization, they form compact and nonconducting MIP films, thus fulfilling the most important criteria for preparation of capacity chemosensors. Furthermore, the insulation property of these films can simply be improved by using SAMs. A chemosensor featuring an MIP recognition unit prepared by electropolymerizing functional and cross-linking monomers, combined with PM transduction, effectively detected analytes with remarkable selectivity and low LOD even in the gas phase, which is not very common in MIP-based chemosensing.

In conclusion, the scope of use of electroactive functional monomers for preparation of MIP films as recognition units of chemosensors is enormous. These monomers have shown their suitability for fabrication of chemosensors using different transduction platforms. They effectively transduce an event of analyte binding, improving the detectability. For higher selectivity of the electrosynthesized MIP, these electroactive functional monomers should be derivatized with additional functional groups. That way, one can justify the choice of a given functional monomer for imprinting with the help of computational modeling.

## Abbreviations

1-NS: 1-Naphthalenesulfonate
2-AThPh: 2-Aminothiophenol
4-AThPh: 4-Aminothiophenol
AA: Ascorbic acid
ACN: Acetonitrile
AMA: 4-Aminobenzoic acid
ANI: Aniline
APBA: 3-Aminophenylboronic acid
APh: 2-Aminophenol
AspA: Aspartic acid
ATP: Adenosine 5′-triphosphate
AuNP: Au nanoparticle
CPF: Chlorpyrifos
CSA: Camphorsulfonic acid
CV: Cyclic voltammetry
DA: Dopamine
DCPA: 2,4-Dichlorophenoxyacetic acid
DHP: *O*,*O*-Dimethyl-α-hydroxylphenyl phosphonate
DMF: *N*,*N*-Dimethylformamide
DMP: Dimethylmethylphosphonate
DPV: Differential pulse voltammetry
DS: Dodecyl sulfate
ECP: Electronically conducting polymer
EDOT: Ethylenedioxythiophene
EDTA: Ethylenediaminetetraacetic acid
EIS: Electrochemical impedance spectroscopy
GA: Glutamic acid
GCE: Glassy carbon electrode
LOD: Limit of detection
MBI: 2-Mercaptobenzimidazol
MIOPPy: Molecularly imprinted overoxidized polypyrrole
MIP: Molecularly imprinted polymer
MIPEDOT: Molecularly imprinted polyethylenedioxythiophene
MIPMG: Molecularly imprinted poly(methylene green)
MIPPD: Molecularly imprinted poly(1,2-phenylenediamine)
MIPPh: Molecularly imprinted polyphenol
MIPPy: Molecularly imprinted polypyrrole
MV^2+^: Methyl viologen
NP: Nanoparticle
OPPy: Overoxidized polypyrrole
OTA: Ochratoxin
OTC: Oxytetracycline
PANI: Polyaniline
PAPBA: Poly(aminophenylboronic acid)
PBS: Phosphate-buffered saline
PD: 1,2-Phenylenediamine
PEDOT: Polyethylenedioxythiophene
PGE: Pencil-graphite electrode
Ph: Phenol
Phe: Phenylalanine
PM: Piezoelectric microgravimetry
PSS: Poly(styrene sulfonate)
PTh: Polythiophene
Py: Pyrrole
PPy: Polypyrrole
SAM: Self-assembled monolayer
SAW: Surface acoustic wave
SCE: Saturated calomel electrode
SPR: Surface plasmon resonance
SWV: Square wave voltammetry
T-2: T-2 toxin
TAA: 3-Thiophene acetic acid
TCAA: Trichloroacetic acid
Th: Thiophene
TL: Tolazoline
TNT: 2,4,6-Trinitrotoluene
Trp: Tryptophan
